# Immunotherapy with Canarypox Vaccine and Interleukin-2 for HIV-1 Infection: Termination of a Randomized Trial

**DOI:** 10.1371/journal.pctr.0020005

**Published:** 2007-01-26

**Authors:** Kendall A Smith, Sofija Andjelic, Zoran Popmihajlov, Liza Kelly-Rossini, Aquanette Sass, Martin Lesser, Steven Benkert, Cory Waters, Joyce Ruitenberg, Paul Bellman

**Affiliations:** 1 The Division of Immunology, Department of Medicine, Weill Medical College of Cornell University, New York, New York, United States of America; 2 General Clinical Research Center, Weill Medical College of Cornell University, New York, New York, United States of America; 3 Investigational Pharmacy, New York Presbyterian Hospital, New York, New York, United States of America; 4 BD Biosciences, San Jose, California, United States of America; 5 Department of Medicine, Weill Medical College of Cornell University, New York, New York, United States of America

## Abstract

**Objectives:**

To determine whether immunotherapy of chronic HIV-1 infection can prevent or attenuate viremia upon antiviral discontinuation.

**Design:**

This was a Phase II randomized, partially double blinded, 2×2 factorial study of three steps of 12 wk/step. Step I involved four groups: (1) vaccine placebo, (2) vaccine (ALVAC, vCP1452), (3) placebo + interleukin 2 (IL-2), and (4) vaccine + IL-2. Step II involved a 12-wk diagnostic treatment interruption (DTI). Step III involved an extension of the DTI for an additional 12 wk.

**Setting:**

The Weill-Cornell General Clinical Research Center.

**Participants:**

Chronically infected HIV-1 positive adults with undetectable HIV-1 levels and > 400 CD4^+^ T cells/μl.

**Interventions:**

An HIV canarypox vaccine (vCP1452) and vaccine placebo, administered every 4 wk for four doses, and low-dose IL-2 administered daily for 12–24 wk.

**Outcome measures:**

Primary endpoints: (1) Proportion of participants with undetectable plasma HIV RNA during trial Step II, (2) mean log_10_ HIV RNA copies/ml ([HIV]) from weeks 21–25, and (3) proportion of individuals eligible for trial Step III.

**Results:**

44 participants were randomized, but 16 withdrew or were withdrawn before completing Step II. As all participants underwent viral relapse in Step II, the study was terminated after 28 participants completed Step II. Among the four groups, there was no difference in mean [HIV] or the proportion of individuals with < log_10_ 4.48 HIV; no difference between the mean [HIV] of the two groups that received ALVAC (*n* = 17) versus placebo (*n* = 11); and no significant difference between the mean [HIV] of the two groups that received IL-2 (*n* = 11) versus placebo (*n* = 17).

**Conclusions:**

Neither ALVAC (vCP1452) nor low-dose daily IL-2 nor their combination prevented the relapse of viremia upon discontinuation of antiviral therapy.

## Editorial Commentary

**Background:** Currently, providing that an individual infected with HIV can get access to highly active antiretroviral therapy (HAART), prognosis can be very good. However, although these treatments suppress replication of HIV, they do not eliminate HIV entirely. One current area of HIV research focuses on whether and how it is possible to take a break from antiretroviral therapy—which is very toxic—and at the same time achieve very low levels of HIV. Some research groups are also interested in whether and how it might be possible to eliminate the virus completely from the bloodstream and immune system of infected people, and therefore achieve a long-term cure. One such approach involves administration of interleukin-2 (IL-2, a hormone involved in the immune response), in the hope that IL-2 will boost the ability of the body's own immune system to eliminate HIV. Various candidate HIV vaccines have also been designed that, it is hoped, will prime the immune system to make it more receptive to IL-2. In the trial reported here, the researchers wanted to test whether IL-2 administration either alone or in combination with an HIV vaccine, ALVAC vCP1452, would maintain low levels of HIV in the blood of people who had stopped taking antiretroviral drugs. Therefore, participants in the trial were assigned to one of four arms: a placebo version of the vaccine only; vaccine only; placebo version of vaccine plus IL-2; or vaccine plus IL-2. The investigators planned to recruit 92 people into the trial who would be given the various interventions tested here for 12 weeks, while also receiving HAART (step 1). Then, HAART was stopped for 12 weeks (step 2), or, in some people, for 24 weeks if HIV levels remained below a certain threshold (step 3).

**What the trial shows:** 44 people were recruited into the study, but it was terminated once results were analysed from those individuals who had completed 12 weeks without HAART. The researchers did not see any significant differences between the treatment groups in any of the three primary outcome measures, which were the proportion of people with undetectable HIV levels during step 2; the average HIV levels during the last four weeks of step 2; and the proportion of people eligible to continue to step 3. Therefore, the investigators concluded that neither the ALVAC vaccine nor IL-2 alone or in combination with each other, prevented HIV from replicating when HAART was stopped.

**Strengths and limitations:** In the trial, randomized assignment to the different treatments was performed by the dispensing pharmacist, so study investigators were not able to predict which treatments the next participant would receive. A further strength in the design of this trial is the use of a placebo vaccine to control for ALVAC vCP1452, which enabled participants and investigators to be blinded to whether a patient received vaccine or placebo. However, a placebo was not used as a control for IL-2 because patients often experience characteristic side effects to this treatment. Limitations in the design of this study include the small numbers of participants, which means that the trial did not have enough power to exclude the possibility of a small effect of IL-2 or vaccine on HIV levels in blood. The follow-up in the trial was also short, and efficacy outcomes did not include measures that patients might consider important, such as survival.

**Contribution to the evidence:** Currently, large-scale trials are evaluating the clinical effectiveness and safety of IL-2 at higher doses than those tested here, together with antiretroviral drugs as treatment for HIV. This study adds data suggesting that IL-2 at a lower dose either with or without the ALVAC vCP1452 vaccine does not prevent HIV replication once antiretroviral drug treatment is stopped.

## INTRODUCTION

Highly active antiretroviral therapy (HAART) is effective in suppressing viral replication in HIV infection, but the infection cannot be eradicated, as evidenced by a rapid return of viremia upon discontinuation of antiretrovirals [1–3]. Therefore, the paramount question now before the HIV community is whether it is actually possible to cure this infection, and if so how [4]? Some suggest that the immune system has already suffered irreparable damage by the time the infection is first diagnosed, thereby precluding complete elimination of the virus. This notion served as the rationale for treating with HAART as soon as possible after infection [5,6]. Unfortunately, by the time the infection is recognized, usually by the onset of systemic constitutional symptoms, several weeks have elapsed and the infection is already widespread. Thus, even the early institution of HAART has failed to effect a cure [7].

Fortunately, there are some infected individuals, albeit rare, who are able to contain the virus and maintain undetectable plasma HIV concentrations for years without any detectable deterioration of immune function [8,9]. These individuals, termed long-term nonprogressors (LTNPs), have come under intense study by immunologists, who have compared the characteristics of the immune systems of LTNPs with the systems of individuals termed “progressors,” who have persistent plasma HIV concentrations and slowly progressive deterioration of immune function. Of all of the immunological tests performed on LTNPs versus progressors, the consistent findings among progressors has been a diminution of circulating CD4^+^ T cells, as well as a deficiency of interleukin 2 (IL-2) production by T cells in response to stimulation by HIV antigens in vitro [10–13]. One consequence of the inability to produce IL-2, which is the predominant T cell growth factor for mature peripheral T cells [14], is a deficient T cell proliferative capacity, and early in the epidemic this was one of the first defects demonstrable in vitro in T cells from HIV-infected individuals [15,16]*.*

In light of these considerations, we performed a preliminary clinical trial to test the capacity of low-dose daily IL-2 administration to boost the immune function of individuals chronically infected with HIV, the results of which were reported in 2000 [17]. Eligible individuals had responded to HAART and had undetectable plasma HIV concentrations (below 50 HIV RNA copies/ml, or [HIV]), as well as CD4^+^ T cell concentrations above 400 cells/μl. To test the ability of the host immune defenses to control viral replication, we continued daily low-dose IL-2 administration, but interrupted HAART for a minimum of eight weeks (termed a “diagnostic treatment interruption” [DTI]), eventually in a total of 15 participants. The phrase “analytical treatment interruption” has also been used to distinguish this approach from a “structured treatment interruption,” which was used as a therapeutic attempt to allow a short-term viral relapse to serve as an endogenous immunization with the individual's own virus.

We found that all participants became viremic rapidly and underwent a peak viremia, with a subsequent 10-fold decline in plasma virus to a trough. Coincident with the reappearance of viremia, but lagging behind the peak plasma virus concentration by about two weeks, was a doubling in the concentration of CD8^+^ T cells. As well, the mean circulating CD8^+^ T cell concentration remained elevated as the peak plasma HIV concentration declined to the lower, more stable trough concentration. By comparison, the circulating CD4^+^ T cell concentration transiently decreased only about 25%, soon after the peak in viremia, while the circulating natural killer cell concentration remained elevated due to the IL-2 administration, and unchanged throughout the eight-week DTI.

Based on these results [16], it appeared that the DTI approach could be used to design a new trial to test immunotherapeutics to boost immune reactivity to HIV. Because low-dose daily IL-2 administration in and of itself had not prevented the viral relapse, we reasoned that a more rational approach to immunotherapy would be to introduce a candidate HIV vaccine to activate the T cell expression of IL-2 receptors, so as to render these cells responsive to the growth-promoting, differentiative, and survival effects of IL-2 before the DTI [14,18,19].

Of the candidate HIV vaccines available for testing, ALVAC, a vaccine produced by Sanofi Pasteur using the canarypox virus as vector, had received the most extensive safety and efficacy testing in field trials in HIV-negative, healthy volunteers [20–23]. No severe reactions (grade III or grade IV) had been observed that were attributable to the vaccine. Thus, the second-generation vaccine, vCP1452, which contains the clade B HIV-1 *env* and *gag* genes and a synthetic polypeptide encompassing the known human CTL epitopes from the *nef* and *pol* gene products, was chosen for testing for the first time in HIV-positive individuals. This vector also contains two vaccinia virus-derived genes, *E3L* and *K3L,* which enhance the overall efficiency of antigen expression.

This report details the interim results from our trial, in which we tested vCP1452 with and without the daily administration of IL-2 as a vaccine adjuvant in a factorial design, and with a DTI as the basis for the clinical trial end point. The rationale followed from our preliminary study [17], which indicated that promotion of a T cell HIV-specific immune response may prevent or attenuate a viral relapse upon discontinuation of antivirals.

## METHODS

### Participants

Individuals eligible for study were chronically infected (i.e., > 6 mo) adults (aged ≥ 18 y) with a positive HIV serology who had never had a CD4^+^ T cell concentration below 200 cells/μl, and who were on HAART (defined as a combination of at least three effective antiretroviral agents), with undetectable plasma HIV concentrations (< 50 copies/ml) and circulating CD4^+^ T cell concentrations 400 cells/μl or above on two occasions 14 d apart within 30 d of enrollment. Individuals were excluded if there was a history of hypersensitivity to any of the study agents, or to eggs and/or birds, especially canaries. Prior exposure to IL-2 must have terminated at least 30 d prior to study entry. Additional exclusion criteria included any prior receipt of an HIV vaccine, a current AIDS-defining illness, documentation of virologic failure (i.e., >10,000 [HIV]) while receiving the current HAART regimen, or a history of thyroid disease, or autoimmune disorders, including asthma, inflammatory bowel disease, rheumatoid arthritis, or psoriasis. Participants were recruited via radio, TV, and print advertisements, as well as via referrals from their primary care physicians. All participants signed a written informed consent form approved by the Weill Medical College of Cornell University Institutional Review Board, which also reviewed the consent form and protocol to ensure that the investigation was conducted according to the principles expressed in the Declaration of Helsinki.

### Objectives

This study was designed to test the hypothesis that immunological enhancement during maximal viral suppression by antiviral drugs can boost the host defenses reactive with the virus, so that upon cessation of antiviral therapy, the plasma virus concentration would remain below detectable levels. Alternatively, should viremia recur, it was hypothesized that immunotherapy would maintain the plasma virus concentration at lower levels than in the absence of immunological enhancement. A secondary objective was to test the hypothesis that the concentrations of circulating HIV p55 *gag*-specific T cells would correlate with control of plasma virus concentrations.

### Interventions

This was a phase II, randomized, controlled, partially double-blinded, single center, 2×2 factorial three-step study to test whether immune-based therapies consisting of an HIV canarypox vaccine and/or daily low dose IL-2 administration provided augmented host antiviral activity, as tested by monitoring plasma HIV concentrations upon cessation of HAART.

Participants were randomized, doubly blinded as to vaccine versus placebo, to one of four groups in Step I as follows: (1) vaccine placebo; (2) vaccine; (3) vaccine placebo + IL-2; and (4) vaccine + IL-2.

During Step I, all participants continued to receive HAART, and in addition received four intramuscular inoculations of vCP1452 vaccine (10^6.5^ TCID_50_) or placebo (vaccine excipient, 0.5 ml) at weeks 0, 4, 8, and 12. The study investigators inoculated vaccine and/or placebo into alternating deltoid muscles.

Those participants randomized to receive IL-2, self-administered daily subcutaneous low-dose (1.2 million U [80 μg]/M^2^ BSA) IL-2 as Proleukin (Chiron Corporation, http://www.chiron.com) injections at sites (abdomen, thighs) distinct from the intramuscular vaccine and/or placebo inoculations throughout the entire study interval of 37 wk, taking care not to inject into the same area on consecutive days because of the known local injection-site delayed-type hypersensitivity reaction elicited by IL-2 [24,25]. Because of this reaction at the site of IL-2 injection, it was considered impossible to blind the IL-2 administration, so it was given open label.

To ensure accuracy of the IL-2 dose administered, the investigational pharmacist at The New York Presbyterian Hospital prepared syringes, each containing a daily dose for 14 d. Each Proleukin vial containing 1.2 mg and 22 million U of IL-2 activity (specific activity = 18.3 million U/mg) was reconstituted with 1.2 ml of sterile water, then diluted further with 4.8 ml of 5% dextrose in water to a final volume of 6.0 ml (specific activity = 3.67 million U [200 μg]/ml). Thus, for an average adult of 1.7 M^2^ BSA, the daily dose was 2 million U (136 μg) in a volume of 0.54 ml. Had the contents of the vial not been diluted, the volume necessary to inject only 2 million U (136 μl) would have been too small to measure accurately in a 1 ml syringe.

At the end of Step I, participants who met eligibility criteria entered Step II, i.e. the DTI, when HAART was discontinued for a minimum of 12 wk. During Step II, as a safety precaution participants were declared off-protocol at any time if the circulating CD4^+^ T cell concentration fell below 250 cells/μl or 50% or less of the mean baseline CD4^+^ T cell concentration on two successive determinations. At the end of Step II, participants were declared off-protocol if the mean plasma [HIV] was 30,000 copies/ml or over during the last 5 wk of study (i.e., weeks 21–25). If the mean plasma [HIV] remained below this threshold, the participants were permitted to remain on study and entered Step III, which consisted of a continuation of the DTI for another 12 wk. During this interval, if the HIV or CD4^+^ T cell thresholds were surpassed on two successive determinations, the participant was declared off protocol. At the end of the Step III (week 37), all participants were declared off-protocol and IL-2 administration was discontinued. When a participant completed the protocol at either the end of Step II or Step III, decisions regarding future HIV medications reverted to the participant, in consultation with the appropriate health care provider.

### Concomitant Medications

During Step I, adjustments or changes in antiretroviral drugs were permitted if for reasons other than virologic failure, such as for ease of administration or because of side effects. Topical corticosteroid use was acceptable, provided it was applied to a site separate from IL-2 and vaccine injection sites. Maintenance therapy for opportunistic infections that developed on study treatment was permitted according to standard medical care, except for foscarnet during IL-2 administration, and rifabutin and rifampin at any time. Other permitted medications were maintenance therapy for recurrent genital herpes with 1,000 mg/day or less of acyclovir, erythropoietin and G-CSF, all antibiotics for bacterial infections as clinically indicated, medications for symptomatic treatment such as antipyretics and analgesics (ibuprofen and acetaminophen were the preferred agents), and concomitant use of thyroid hormone replacement to manage hypothyroidism.

The following drugs were excluded during study: interferons, other interleukins, other HIV vaccines, sargramostim (GM-CSF), dinitrochlorobenzene (DNCB), thymosin alpha 1 (thymosin alpha), thymopentin, inosiplex (Isoprinosine), polyribonucleoside (Ampligen), ditiocarb sodium (Imuthiol), investigational antiretroviral agents, thalidomide, St. John's wort, systemic or local cytotoxic chemotherapy for malignancy, and systemic corticosteroids.

### Schedule of Evaluations

During Step I, clinical and laboratory evaluations were performed prior to each vaccine or placebo inoculation at 4-wk intervals. During Step II, clinical and laboratory evaluations were performed weekly, and during Step III, clinical and laboratory evaluations were obtained bimonthly. All clinical observations were recorded in a secure electronic database. Laboratory data were transferred electronically daily from the New York Presbyterian Hospital Laboratory computer to the electronic database.

### Laboratory Evaluations

Lymphocyte subset concentrations were monitored by the New York Presbyterian Hospital Laboratory using standard methods for performing flow cytometry. HIV-specific cytokine-producing lymphocyte precursor (CPLp) concentrations were determined after a short-term in vitro activation with a 15-mer peptide pool overlapping every 11 amino acids predicted by the entire coding region of the HIV p55 *gag* gene sequence. See Text S1 for details.

### Outcomes

Antiretroviral therapy was interrupted for a minimum of 12 wk during Step II, and three primary endpoints were used for evaluating immune control of viral replication, based upon plasma [HIV] (see Text S1); (1) the proportion of participants who had undetectable plasma [HIV] during Step II, (2) the mean of the log_10_ [HIV] obtained from weekly blood samples during on-study weeks 21–25, which corresponded to weeks 8–12 following the cessation of HAART, and (3) the proportion of participants who were eligible to progress to Step III, as determined by mean plasma [HIV] of (< 30,000 [log_10_ 4.48] copies/ml during the last five weeks of Step II (i.e., weeks 21–25). This criterion was based on our preliminary data from an addition of six participants to the nine reported originally [16] who received daily low dose IL-2 during an 8-wk DTI. The data from this group of 15 participants revealed that there were 8/15 volunteers who had a mean trough plasma [HIV] below 30,000 (log_10_ = 4.48). This group had a mean ± standard deviation of 3.77 ± 0.27 copies/ml, whereas 7/15 volunteers who had a mean trough plasma [HIV] > 30,000 (log_10_ = 4.48), had a mean of 4.82 ± 0.31 copies/ml. The difference between the means of the two groups was significant (*p* < 10^−4^, two-tailed Student's t-test).

### Sample Size

It was calculated that 23 participants/group would be required, assuming a dropout rate of 25%, resulting in 17 evaluable patients per arm. Based on our previous open label study using daily low-dose subcutaneous IL-2 administration both before and after the cessation of HAART, the mean plateau value ± standard deviation of the “trough” plasma [HIV] of nine participants was 4.2 ± 0.5 log_10_. Using a Bonferroni-adjusted t-test approach to the power calculation for comparing each of the three active treatment arms to the vaccine placebo control group (alpha = 0.05/3 = 0.017), this yielded 90% power for detecting a difference of 0.65 log units of the mean plasma [HIV].

### Randomization

The investigational pharmacist assigned participants to receive IL-2 or not, and to receive ALVAC or not, according to a computer-generated randomization procedure. A balanced selection process consisted of 11 blocks of four participants each, and resulted in the assignment of 11 participants to each of the four treatment groups. The random allocation sequence was concealed from the study investigators and participants via a telephone call to the pharmacist. After assignment to a treatment group by the pharmacist, the study investigators were informed as to whether the participant would receive IL-2 or not. The pharmacist then furnished syringes containing the first 14-d supply of IL-2, along with a syringe containing vaccine or placebo for administration by the study investigator.

### Blinding

Participants, study investigators, and laboratory personnel were all blinded to vaccine or placebo group assignment. Neither the vaccine nor placebo caused a local reaction, so it was not possible for either the study investigators or the participants to discern whether vaccine or placebo was administered. The IL-2, in contrast, was administered in open-label fashion because it caused a local inflammatory reaction at the site of administration.

### Statistical Methods

Mean log_10_ plasma [HIV] values were compared using ANOVA for a 2×2 factorial design. Initially, the vaccine × IL-2 interaction term was evaluated in order to determine whether the difference in viral load response between vaccine and placebo control varied according to whether or not IL-2 was administered. If the interaction was significant, then specific “within level” contrasts were performed. A Fisher exact test was used to compare proportions between the four experimental groups.

A second set of statistical analyses focused on the behavior of circulating CD4^+^ and CD8^+^ cell concentrations during Step II (weeks 13–25) as compared to the mean of three baseline determinations (100%). Repeated measures analysis of variance, or RMANOVA, using the mixed model approach (SAS PROC MIXED) was applied to each of these parameters to determine the effects of time, IL-2, and vaccine. Terms for the interaction between IL-2 and vaccine, as well as for time were included. In addition, the mean plasma log_10_ [HIV] from the last 5 wk of Step II (weeks 21–25) were computed and compared with the mean circulating CD4^+^ and CD8^+^ T cell concentrations for each person individually and then for the group eligible for Step III and the group ineligible for Step III. Then the Pearson correlation coefficients between the mean plasma log_10_ [HIV] and mean CD4^+^ and CD8^+^ T cell concentrations were computed.

A repeated measures ANOVA using the mixed model was used to examine changes in circulating HIV *gag* p55-specific CPLp concentrations over three time intervals in the study (i.e., background [week 0], at the end of Step I [week 13], and during the viremic phase of Step II [week 21]), and across treatment groups (i.e., A, B, C, and D). A group × time interaction term was included in the model and a compound symmetry covariance structure was assumed. A result was considered statistically significant if *p* < 0.05.

### Interim Analysis

An interim analysis was planned for efficacy, after half of the projected sample size was followed for the required 25 wk. Thus, the interim analysis was planned when 34 participants completed Step II (calculated based upon a dropout rate of 25%, yielding 92 enrolled participants × 75%/2 = 34). At that time, the 2×2 factorial ANOVA was to be used. Using a Lan-DeMets version of an O'Brien-Fleming group sequential stopping rule, the trial was to be stopped if at least one of the three tests (i.e., main effect for vaccine, main effect for IL-2, or interaction) was statistically significant at the alpha = 0.00305 significance level. If none of the tests was significant, then the trial was to continue until the planned termination with 92 individuals enrolled and 68 had completed Step II. The final ANOVA was planned to be carried out at the alpha = 0.04695 significance level, to preserve the projected statistical powers.

## RESULTS

### Participant Flow

**Figure 1:**
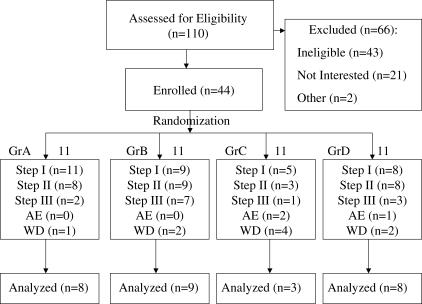
Participant Flow Each box represents the flow of participants through the study, from screening to enrollment to randomization and analysis. AE, adverse event necessitating withdrawal from study; WD, individual withdrew from study (see text).

Between December 2000 and February 2005, 110 individuals were screened for eligibility. Of these, 66 (60% of total) were excluded: 43 (65%) were ineligible, 21 (32%) were not interested, and two (3%) did not meet the study entry criteria according to the opinion of the investigators. Of the 44 participants enrolled (40% of total), 11 were randomly assigned to each experimental group (Figure 1). In group A, three participants were terminated according to protocol during Step II because of low CD4^+^ T cell concentrations. Of the eight participants who completed Step II, five were ineligible to continue to Step III because of plasma [HIV] in excess 30,000 copies/ml. One participant who was eligible for Step III withdrew from the study rather than continue the DTI. In group B, one participant withdrew consent before receiving study medications, and one participant withdrew consent during Step I because he/she wanted to discontinue HAART immediately and not wait for Step II. Of the nine participants who finished Step II, seven were eligible for Step III, whereas two participants were not eligible due to plasma [HIV] over 30,000 copies/ml. In group C, four participants withdrew during Step I because of intolerance to IL-2 injections, one withdrew during Step II because he/she wanted to restart HAART, two were terminated because of the development of hyperthyroidism, and one was terminated because of low CD4^+^ T cell concentrations. Of the three participants who finished Step II, only one was eligible for Step III. In group D, two participants withdrew during Step I because of intolerance to the IL-2 injections, and one was terminated because of the development of antibodies reactive with Proleukin. Of the eight participants who completed Step II, only three were eligible for Step III.

Of the 44 participants enrolled to the study over 44 months, 28 (65%) completed Step II and were evaluable at the time of the interim analysis. Of these 28 participants, 14 qualified for Step III, and only one of 14 elected not to continue to enter Step III.

In view of a viral relapse in all participants during Step II, which was the first primary endpoint, as well as the slow rate of enrollment of about one participant per month, and the higher than expected termination rate (i.e., 36% observed versus 25% expected), with agreement by the study sponsors it was elected to perform an interim analysis after 28 rather than 34 participants had completed Step II and were therefore evaluable.

### Baseline Data

Between May 2001 and November 2004, 44 participants were enrolled. Enrolled participants attended two screening visits and three baseline visits from which baseline data were obtained. There were three females and 41 males. The average age of volunteers was 41 y (range 22–63 y). The mean ages of participants randomized to the four treatment groups were similar (Table 1). Also listed are the mean lymphocyte subset concentrations. Within the total participant population, the mean ± standard error of the mean (SEM) CD4^+^ T cell concentration was 648 ± 28 cells/μl, while the mean CD8^+^ T cell concentration was 891 ± 56 cells/μl. As shown in Table 1, there was not a significant difference of the T cell subset concentrations among the four treatment groups.

**Table 1:**
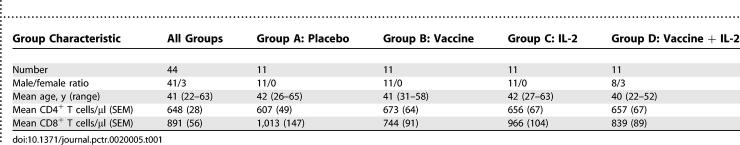
The Demographics of the Study Participants Showed Equal Distribution among All Treatment Groups

### Outcomes and Estimation: Primary Outcomes

#### Viral dynamics.

**Figure 2:**
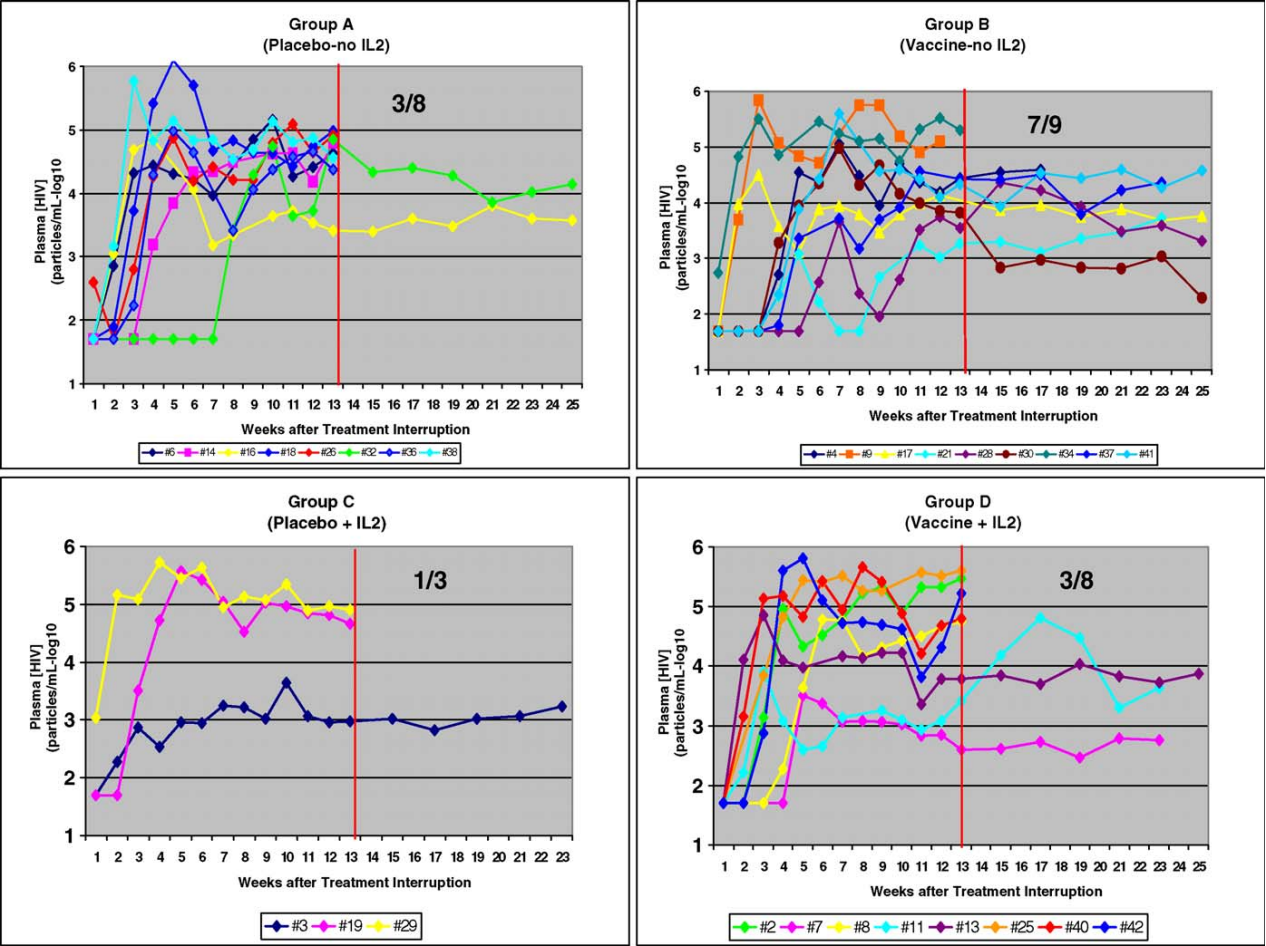
Plasma [HIV] during Steps II and III of Individuals According to Immunotherapy Treatment Group Red vertical line in center of each figure depicts the demarcation between Step II and Step III. Fractions in upper right corner of each figure portray the number of participants who were eligible to enter Step III in each group.

The plasma [HIV] of all participants remained undetectable throughout Step I. Figure 2 shows the viral dynamics for each participant according to study group during Step II and Step III. It is evident from these plots, that all individuals relapsed in Step II after the discontinuation of HAART (the first primary endpoint), and the onset of detectable viremia was variable, ranging from 1 to 7 wk. The peak plasma [HIV] ranged from 3 to 6 log_10_, and the plasma [HIV] of each participant reached the plateau phase during the last 5 wk of Step II (study weeks 21–25), i.e., 8–12 weeks after the antiviral treatment interruption (the second primary endpoint). Thereafter, those participants eligible to proceed to Step III (the third primary endpoint) maintained stable plasma [HIV] throughout the additional 12-wk Step III interval (i.e., study weeks 25–37).

**Figure 3:**
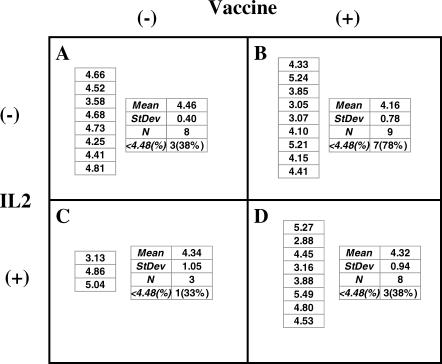
Results from Interim Analysis This was a 2×2 factorial study with participants randomized to receive vaccine (+) or not (−), and IL-2 (+) or no-IL-2 (−). The numbers listed in each block are the mean plasma [HIV] log_10_ RNA copies/ml for each participant from the last 5 weeks of Step II (i.e., weeks 21–25). There was no significant difference between the means of each group based on a 2×2 ANOVA.

Analysis of the mean plasma [HIV] in log_10_ copies/ml of the four groups during the last 5 wk of Step II (the second primary endpoint) were not significantly different when compared with one another (Figure 3). Based on a 2×2 ANOVA there was no effect of vaccine (*p* = 0.62), nor IL-2 (*p* = 0.94), nor a synergistic or antagonistic effect between the vaccine and IL-2 as evidenced by the lack of a significant interaction (*p* = 0.68). As well, the percentage of participants with plasma [HIV] below 30,000 (< log_10_ 4.48) copies/ml varied from 33% to 78% among the separate treatment groups (the third primary endpoint), but again these differences were not significant based on a Fischer exact test. In this regard, it is important to emphasize that the number of participants in each experimental group would not have been predicted to approach significance, based upon our study design.

However, based on these results, and in view of the differential termination rate among the four groups, it was elected to unblind and terminate the study. Accordingly, it was determined that group A received the vaccine placebo; B, vaccine; C, placebo + IL-2; and D, vaccine + IL-2 (Figure 3). Because of the differential termination rate with more participants terminated from groups C and D than from groups A and B, group C (placebo + IL-2) had only three participants evaluable.

### Outcomes and Estimation: Secondary Outcomes

#### Lymphocyte dynamics.

**Figure 4:**
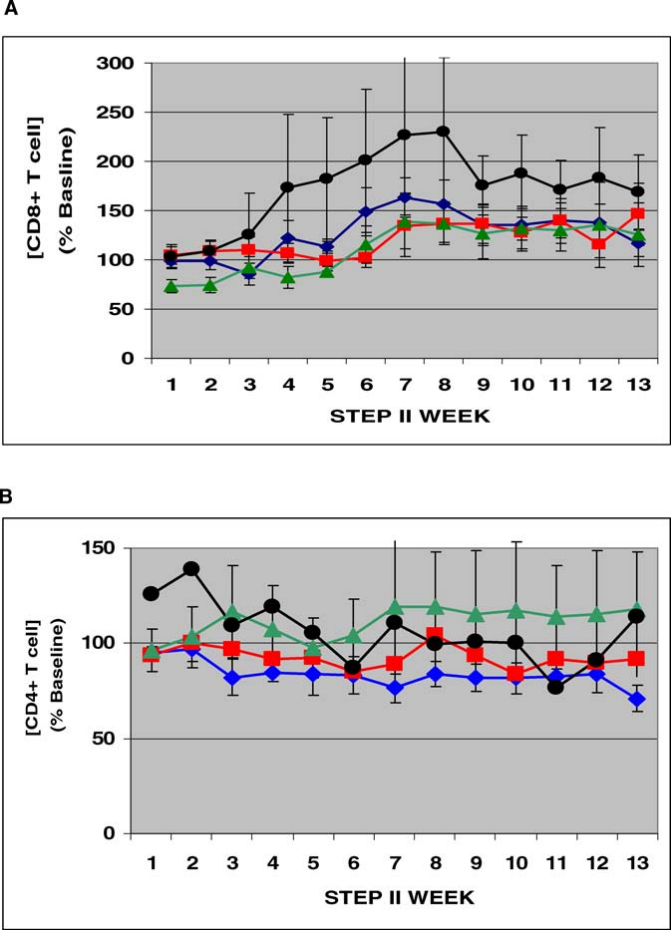
Mean CD8^+^ and CD4^+^ T cell Concentrations of the Four Immunotherapy Groups during Step II (A) CD8^+^ T cells. (B) CD4^+^ T cells. Blue diamonds = group A, placebo; red squares = group B, vaccine; green triangles = group C, IL-2; black circles = group D, IL-2 + vaccine. Brackets depict SEM.

In the context of CD8^+^ T cell concentrations from each experimental therapy group during Step II, there was a significant interaction between IL-2 and vaccine. In the absence of IL-2 administration, the use of vaccine (group B) did not result in a significant increase in circulating CD8^+^ T cell concentrations compared with placebo (group A) (*p* > 0.3). However, the circulating CD8^+^ T cell concentrations were increased significantly when IL-2 was coadministered with vaccine (group D) (*p* < 0.002) (Figure 4A).

For CD4^+^ T cell concentrations, statistical analysis showed no interaction between IL-2 and vaccine, but there was a main effect of IL-2 (*p* < 0.0001) and no effect of vaccine. That is, the use of IL-2 was associated with increased concentrations of circulating CD4^+^ T cells, and this increase was statistically similar whether vaccine was administered or not (Figure 4B). In addition, there was a significant negative correlation for the group eligible for Step III between the mean plasma log_10_ [HIV] and the mean CD4^+^ T cell concentration (*r* −0.66, *p* < 0.01).

### Changes in HIV p55 *gag*-Reactive T Cell Concentrations

**Figure 5:**
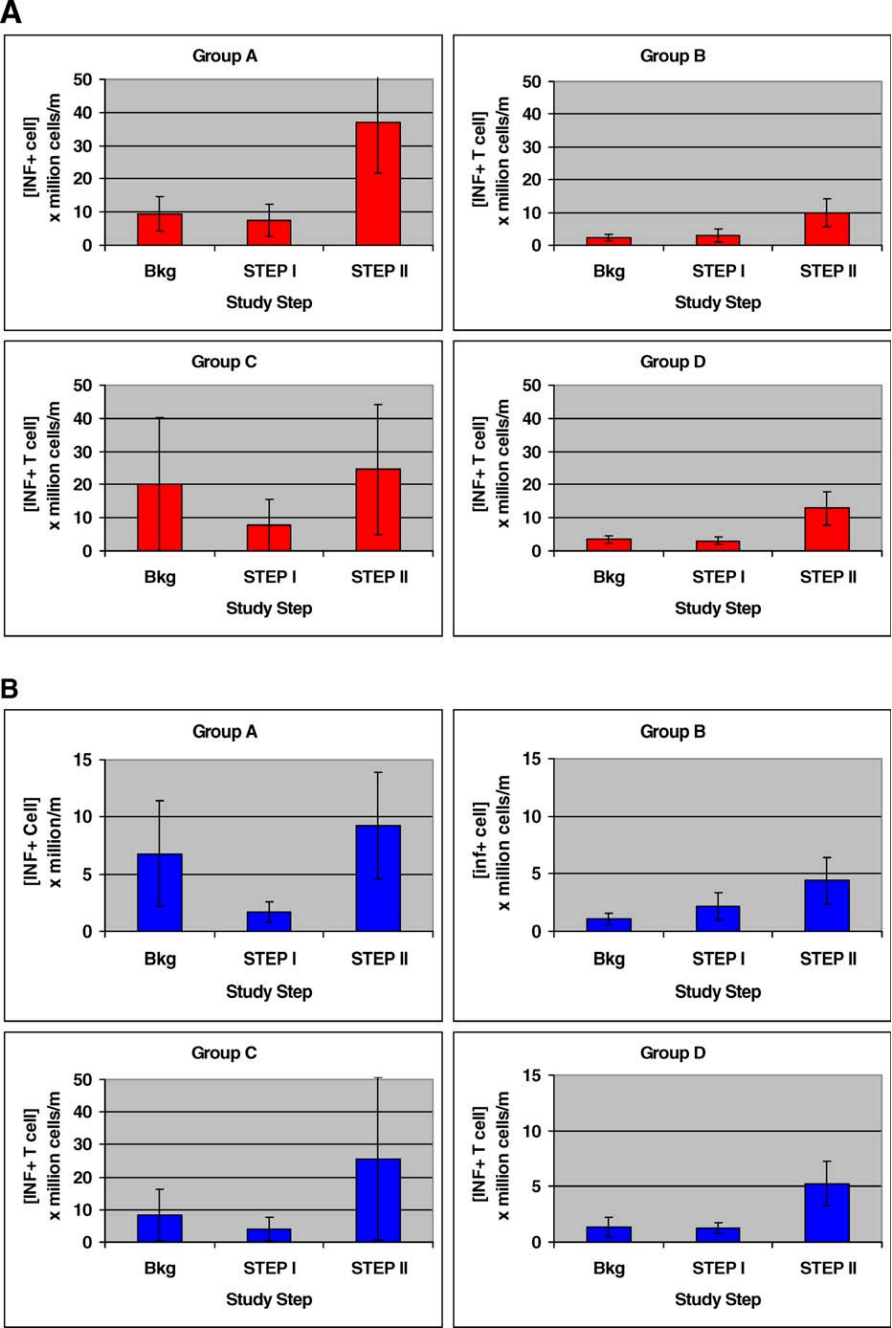
Mean HIV p55 *gag*-Specific T Cells Capable of Producing IFN-γ According to Treatment Group (A) [CD8^+^ T cell]. (B) [CD4^+^ T cell]. Brackets depict SEM. Bkg, background.

The changes in circulating HIV p55 *gag-*specific CD8^+^ T cell concentrations among the four treatment groups as monitored by interferon gamma (IFN-γ)–secreting CPLps are depicted in Figure 5A. There were no significant differences according to treatment group (*p* = 0.21), but the cell concentrations increased significantly at Step II relative to Step I and background (*p* < 0.001). As well, as shown in Figure 5B, there were no significant differences in circulating HIV p55 *gag*-specific CD4^+^ T cell concentrations among the four treatment groups (*p* = 0.20), but the CD4^+^ T cell CPLps increased significantly at Step II relative to Step I and background (*p* < 0.01).

**Figure 6:**
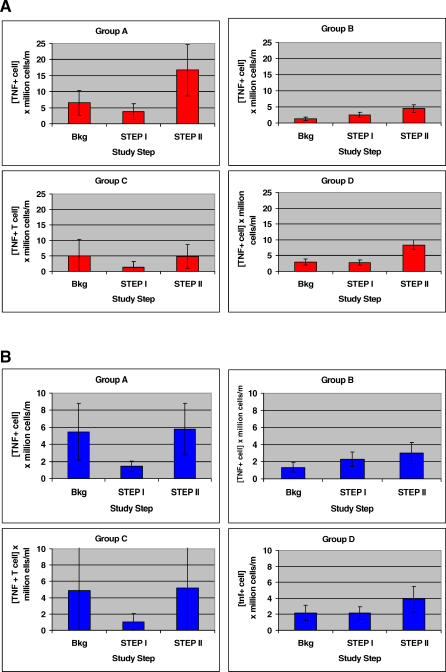
Mean HIV p55 *gag*-Specific T Cells Capable of Producing TNF-α According to Treatment Group (A) [CD8^+^ T cell]. (B) [CD4^+^ T cell]. Brackets depict SEM. Bkg, background.

Similar results were obtained comparing tumor necrosis factor alpha (TNF-α)–secreting CD8^+^ CPLps as shown in Figure 6A. There were no significant differences according to treatment group (*p* = 0.43), but there was a significant increase at Step II relative to Step I and background (*p* = 0.01). By comparison, there were no significant differences in CD4^+^ TNF-α–secreting CPLps comparing the treatment groups (*p* = 0.75) or the time intervals during the study (*p* = 0.09) (Figure 6B).

**Figure 7:**
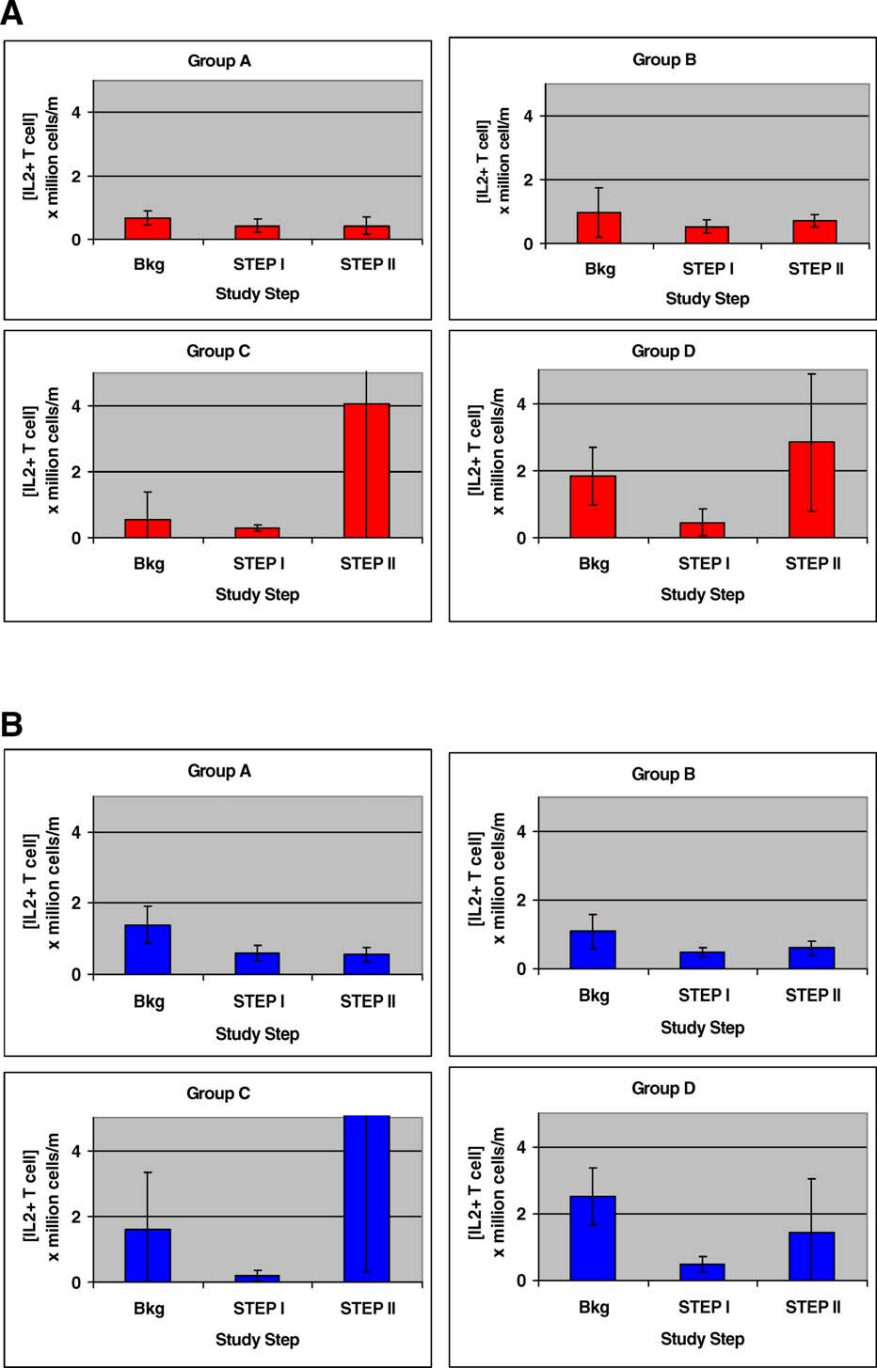
Mean HIV p55 *gag*-Specific T Cells Capable of Producing IL-2 According to Treatment Group (A) [CD8^+^ T cell]. (B) [CD4^+^ T cell]. Brackets depict SEM. Bkg, background.

Compared with the IFN-γ and TNF-α CPLp concentrations, the IL-2–secreting CPLp concentrations were an order of magnitude lower overall (Figure 7). The CD8^+^ IL-2–secreting CPLps were not significantly different comparing treatment groups (*p* = 0.53), or comparing time intervals of the study (*p* = 0.06) (Figure 7A). The CD4^+^ IL-2–secreting CPLp concentrations were not significantly different comparing treatment groups (*p* = 0.52), but there was a significant increase at Step II compared with Step I or background (*p* < 0.03) (Figure 7B).

### Adverse Events

The NIAID Division of AIDS Table for grading Severity of Adult Adverse Experiences (http://rcc.tech-res.com/tox_tables.htm) was used for reporting adverse events. Toxicity was graded on a scale of I to IV, with severe and life-threatening events graded as III or IV, respectively.

A total of 610 AEs were reported; of which 529 (86.7%) were grade I, 77 were grade II (12.6%), and only four (0.7%) were grade III. The grade III AEs consisted of allergy to IL-2 (one, group D), and neutropenia (three—one in group A and two group C). There were no grade IV AEs and there were no serious AEs (SAEs). Table 2 lists the types of AEs higher than grade I, as the number and percent of the total AEs observed, as well as those in each of the four experimental groups. There were no protocol deviations.

**Table 2:**
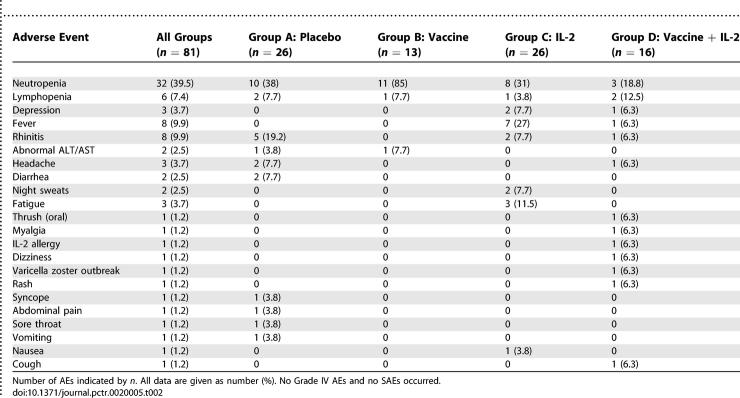
Adverse Events above Grade I

## DISCUSSION

### Interpretation

The results obtained from this Phase II immunotherapy trial for individuals with chronic HIV infection indicate that neither four monthly intramuscular inoculations of ALVAC (vCP1452), nor daily low dose IL-2 subcutaneous injections, nor their combination, can prevent a viral relapse when antiretroviral therapy is discontinued.

Because all participants relapsed when antiretrovirals were discontinued, the study was terminated prematurely. This decision was also influenced by the slow enrollment rate of only one participant per month and a dropout/termination rate greater than anticipated (i.e., 36% versus 25%). Accordingly, additional trials will be necessary to determine whether this immunotherapy protocol can yield benefit by attenuating rather than actually preventing the viral relapse.

The difficulty in enrollment was due to several problems, including (1) it was a randomized study with a chance of one-fourth that a volunteer would be randomized to no experimental therapy, (2) the study involved a treatment interruption, (3) there were frequent clinic visits, (4) the participants who received IL-2 had to self-inject daily, and (5) many physicians and patients were skeptical that immunotherapy would yield any benefit.

With regard to enrolled participants who were discontinued from the study, particularly noteworthy was a differential dropout/termination rate from the groups that received IL-2. Of the 11 participants lost from the IL-2 treatment groups, six dropped out early in the study because they were unable or unwilling to undergo daily self-injections, or because of the mild (grade I) local inflammatory reaction or the mild (grade I) systemic constitutional symptoms associated with low-dose IL-2 administration (i.e., malaise, myalgia, fever). Accordingly, even though the dose of IL-2 was low enough to avoid most of the severe systemic constitutional signs and symptoms of inflammation associated with higher IL-2 doses, it is clear that low-dose IL-2 administration is hampered by the fact that it must be injected daily, and still causes both local and systemic inflammation.

With regard to AEs, it is worthy of mention that only 13% of the AEs were higher than grade I, and there were no grade IV AEs or SAEs. As well, there were no instances of a “retroviral syndrome” severe enough to cause withdrawal from the study. Accordingly, this clinical trial design for healthy HIV-positive individuals on antiretrovirals is well tolerated. It is also noteworthy that there were few AEs attributable to the administration of the vaccine.

### Generalizability

The fact that all participants underwent a viral relapse within a few weeks after antivirals were discontinued was disconcerting in view of the hope that some individuals might mount an immune response effective enough to control viral replication, especially because the total viral burden had been reduced to low levels by antiviral chemotherapy. Our findings argue strongly that additional studies are necessary to more fully understand the failure of the immune system to control viral replication in these patients. In this regard, it is especially disappointing that IL-2 administration had no discernible effect, given the numerous studies, noted previously, that point to a defect in IL-2 production as one of the hallmarks of the immunodeficiency of chronic HIV infection.

The heterogeneity of individuals with regard to the viral dynamics in Step II (Figure 3) should be taken into consideration in the design of future immunotherapy clinical trials. The broad range of peak and plateau plasma [HIV], even in the absence of immunotherapy, is indicative of an individual heterogeneity that will require experimental groups of more than 17 volunteers, as estimated for this study originally, to resolve whether differences observed might be due to experimental therapy.

There was a definite effect of IL-2 administration, which served to increase the concentration of circulating CD8^+^ T cells when coadministered with vaccine (Figure 4A). It is unclear whether the magnitude of the increase can be attributed to an effect of the vaccine, IL-2, or both from these data, because there were only three participants evaluable in the group that received IL-2 alone. However, it is likely that the CD8^+^ lymphocytosis was primarily due to IL-2, in that a similar magnitude of increase was observed in our preliminary trial that did not include vaccine [17]. IL-2 also functioned to maintain the baseline concentrations of circulating CD4^+^ T cells after the discontinuation of the antivirals, at least for the duration of the study. These results suggest that low daily doses of IL-2 might be used to maintain the circulating CD4^+^ T cell concentrations if individuals discontinue antivirals for longer intervals. However, further studies will be required to determine this point.

At the initiation of the study it was hoped that monitoring the frequency of HIV p55 *gag-*specific T cells by flow cytometry would provide an additional parameter to compare with the plateau plasma [HIV]. However, there was no change from baseline after the immunotherapy interval (Step I) in the concentrations of circulating HIV-specific T cells whether monitored by IFN-γ, TNF-α, or IL-2 production. Moreover, even though there was a readily detectable increase in circulating HIV p55 *gag-*specific CD4^+^ and CD8^+^ T cells when monitored after the peak of viremia at week 21 during Step II, there was not a significant difference when comparing the treatment groups. However, these results must be interpreted in light of a lack of difference in the viral endpoints when comparing treatment groups. In addition, the increase in detectable CPLp that occurred during the viremic phase of Step II indicates that the participants were capable of mobilizing increased concentrations of HIV-specific T cells, given an increased antigenic stimulus.

Accordingly, additional studies are warranted to determine whether the CPLp assay or additional in vitro immunological correlates of a favorable in vivo antiviral immune response can be identified. As well, these data indicate that the monitoring of immunological responses should be combined with a plasma [HIV] endpoint to adequately predict any correlation between the in vitro immunological assays and an antiviral immune response. Also, it is possible that a stronger immune response, if elicited during Step I before the discontinuation of antivirals, could serve to prevent the viral relapse.

Perhaps the most consistent finding with regard to the frequencies of cytokine-producing cells was the very low level of detectable IL-2-producing cells regardless of treatment group and throughout the study intervals that were monitored. Moreover, it is noteworthy that IL-2 therapy had no effect on IL-2–producing capacity, a result not entirely unexpected, given the lack of influence of IL-2 on its own gene expression [26,27]. Consequently, future studies are necessary to focus on the IL-2 gene and its regulation to gain additional insight into this immune defect in HIV infection.

### Overall Evidence

The results of this study are consistent with those of two other studies reported recently where the effects of either ALVAC or IL-2, or both, were examined. The ANRS 093 study is perhaps the most favorable study in terms of support for the concept that immunotherapy or therapeutic immunization before antiviral drug cessation may contribute to the capacity of the host to contain HIV replication [28]. The AIDS Clinical Trials Group has completed a trial (#5024), which compared ALVAC (vCP1452) to cycles of high-dose IL-2, versus their combination in chronically infected participants [29]. The investigators concluded that while there was a suggestion that therapeutic immunization might have the potential to enhance host immune control after antiretroviral cessation, the effect is modest (median difference ∼ 0.4 log_10_ HIV RNA copies/ml). Moreover, even high-dose IL-2 did not appear to add benefit to the vaccine.

One must conclude from the results of these studies that the canarypox vaccine, which cannot replicate in mammalian cells, is a relatively weak immunogen. As already pointed out [30], viral vectors that are replication incompetent suffer from the inability to maximally activate antibody formation because mature virions are not produced to stimulate B cell recognition of native envelope structures, nor are opsonized virions available for uptake by antigen-presenting cells for processing and presentation of vaccine epitopes to CD4^+^ T helper cells via class II major histocompatibility complex. Accordingly, replication-incompetent vectors must rely almost entirely on stimulating primarily a CTL response. Unfortunately, almost all of the HIV vaccines currently under development are in replication-incompetent vectors. The one exception is being conducted in a program at the National Cancer Institute, which is developing replication-competent adenovirus as an HIV vaccine vector [31–33]. Their recent results, showing a durable protective CD8^+^ T cell immune response to viral challenges a year apart, provide hope that effective vaccines against HIV will eventually be possible [33].

## SUPPORTING INFORMATION

CONSORT Checklist Click here for additional data file. (54 KB DOC)

Trial Protocol Click here for additional data file. (901 KB PDF)

Text S1 Laboratory Evaluations (27 KB DOC)

## Abbreviations

CPLp: cytokine-producing lymphocyte precursor
DTH: delayed type hypersensitivity
DTI: diagnostic treatment interruption
HAART: highly active antiretroviral therapy
[HIV]: HIV RNA concentration (copies/ml)
IL-2: interleukin 2
IFN-γ: interferon gamma
LTNP: long-term nonprogressor
SAE: serious adverse event
SEM: standard error of the mean
TNF-α: tumor necrosis factor alpha
