# Supplementary material for: Immunotherapy with Canarypox Vaccine and Interleukin-2 for HIV-1 Infection: Termination of a Randomized Trial
Source: PLoS Clin Trials. 2007 Jan 26;2(1):e5. doi: 10.1371/journal.pctr.0020005 (PMC1783674; doi:10.1371/journal.pctr.0020005)
Supplement: Trial Protocol [file pctr.0020005.sd002.pdf]

A RANDOMIZED CONTROLLED TRIAL TESTING THE EFFICACY OF IMMUNOTHERAPIES*TO CONTROL PLASMA HIV RNA CONCENTRATIONS UPON INTERRUPTION OF HIGHLY ACTIVE ANTIRETOVIRAL THERAPY

*** CANARYPOX HIV VACCINE (vCP1452) AND DAILY LOW DOSE INTERLEUKIN 2 ADMINISTRATION**

Sponsoring Institution:

Weill Medical College of Cornell University

Supporting Institution:

The National Institute of Allergy & Infectious Diseases
Grant # RO1 AI51181-01

Pharmaceutical Support Provided by:

Aventis Pasteur
Chiron Corporation
Becton Dickinson

IND # 9402

Principle Investigator & Protocol Chair:

Kendall A. Smith, M.D.

Weill Medical College of Cornell University
1300 York Avenue
New York,
NY
10021

NIAID VERSION 6
December 13, 2003

1 PROTOCOL TEAM ROSTER 4
2 STUDY MANAGEMENT 7
3 SCHEMA 8
4 OBJECTIVE: 9
4.1 Primary Endpoint 9
4.2 Secondary Endpoint: 9
5 INTRODUCTION 11
5.1 Background & Rationale: 11
5.2 Canarypox vaccines (vCP1452, ALVAC) 13
5.2.1 Pre-clinical Studies: 14
5.2.2 Safety in HIV Seronegative Subjects 15
5.2.3 Carcinogenesis, Mutagenesis, Impairment of Fertility: 15
5.2.4 Pregnancy: 16
5.2.5 ALVAC-HIV vCP1452 - to be studied in this protocol 16
5.3 Daily Low-Dose IL2 Administration to HIV+ Individuals 17
5.3.1 Phase I 17
5.3.2 Phase I/II 17
5.3.3 Amgen IL2 vs. Chiron IL2 18
5.3.4 Phase II 18
5.3.5 IL2 Studies to Augment Antigen-Specific Immunity 19
6 STUDY DESIGN 19
7 SELECTION AND ENROLLMENT OF SUBJECTS 20
7.1 Step I: Receipt of Study Medication(s) and Potent Antiretroviral Therapy 20
7.1.1 Inclusion Criteria: Step I 20
7.1.2 Exclusion Criteria: Step I 21
7.1.3 Inclusion of women and minorities: 22
7.1.4 Recruitment 23
7.1.5 Criteria for withdrawal from Step I: Inability to maintain viral control: 23
7.2 Step II: Interruption of Antiretroviral Therapy: 24
7.2.1 Inclusion Criteria 24
7.2.2 Exclusion Criteria 24
7.3 Criteria to terminate Step II 24
7.4 Step III – extension of Interruption of HAART 25
7.4.1 Inclusion 25
7.4.2 Exclusion 25
7.5 Study Enrollment Procedures 25
8 STUDY INTERVENTION 26
8.1 Drug Regimens, Administration, and Duration 26
8.1.1 Timing of ALVAC vCP1452 or ALVAC placebo administration 26
8.1.2 Timing of IL2 administration 26
8.1.3 IL-2 Preparation and Administration: 26
8.1.4 ALVAC vCP1452/ALVAC Placebo Preparation and Administration: 27
8.1.5 Drug Formulation: 27
8.1.6 Drug Supply, Distribution, and Pharmacy 28
8.1.7 Investigational agent accountability 28

8.2 Concomitant Medications: 29
8.2.1 Permitted Medications: 29
8.2.2 Prohibited Medications: 29
8.2.3 Adherence Assessment 30
9 CLINICAL & LABORATORY EVALUATIONS 30
9.1 Schedule of Events: (Please refer to Evaluations Table, Appendix) 30
9.1.1 Screening Visits (SV 1 & 2): 30
9.1.2 Randomization & Baseline Visits (weeks -2, -1, 0) 31
9.1.3 Step I, Vaccination-weeks 4 # 8, and 12: 32
9.1.4 Post vaccination weeks-#s 5, 9 and 13 33
9.1.5 Week 12.5 Phone visit: 33
9.1.6 Week 13: Begin Step II: 33
9.1.7 Weeks 14-16: 33
9.1.8 Week 17): 33
9.1.9 Weeks 18-20), Weeks 22-24) and bi-weekly during Step III(wks 25-36) 34
9.1.10 Weeks 21 & 25 34
9.2 Weeks 26 – 36 Step III 34
9.3 Definitions for Schedule of Events: 35
10 TOXICITY MANAGEMENT 39
10.1 Description and management of adverse reactions 39
10.1.1 Study Treatment Discontinuation for Toxicity 39
10.2 Reinstitution of HAART 41
10.2.1 Low CD4+ T cell Concentrations 41
10.3 Criteria for Study Discontinuation: 42
11 STATISTICAL CONSIDERATIONS 42
11.1 General design Issues 42
11.2 Primary Endpoint: 43
11.3 Sample Size & Power for the Primary Endpoint: 43
11.4 Secondary Endpoints: 43
11.5 Sample Size & Power for the Secondary Endpoints: 44
11.6 Interim Analysis and Early Stopping 44
12 ADVERSE EVENT REPORTING 44
13 DATA COLLECTION/MANAGEMENT 45
13.1 Overview 46
13.2 Database Design and Programming 46
13.3 Data Entry Procedures 46
13.4 Data Quality Assurance: Queries and Audits 47
13.5 Confidentiality 47
13.6 Data Backup Procedures 47
13.7 Hardware and Software 47
14 SAFETY MONITORING 48
15 HUMAN SUBJECTS 48
15.1 Institutional Review Board (IRB) Review and Informed Consent 48
15.2 Subject Confidentiality 49
15.3 Study Discontinuation 49
16 PUBLICATION OF RESEARCH FINDINGS 49

17 BIOHAZARD CONTAINMENT 49
18 REFERENCES.. 50

1 PROTOCOL TEAM ROSTER

Principal Investigator

Kendall A. Smith, M.D.
Weill Medical College
Cornell University
Division of Immunology
Department of Medicine
515 East 71st St.
New York, NY 10021
Phone: 212-746-4608
FAX: 212-746-8167
EM: kasmith@med.cornell.edu

Investigators

Paul Bellman, M.D.
Weill Medical College
Cornell University
Division of Immunology
Department of Medicine
99 University Place
New York, NY
Phone: 212-673-1000
FAX:212-673-0408
EM: paulpcb1@aol.com

Andrea Cerutti, M.D.
Weill Medical College
Cornell University
Department of Pathology
525 East 68th St.
New York, NY 10021
Phone: 212-746-6396
Fax: 212-746-4527
EM: acerutti@med.cornell.edu

:Investigator

Sofija Andjelic, M.D.
Weill Medical College
Cornell University
Division of Immunology
Department of Medicine
515 East 71st St.
New York, NY 10021
Phone: 212-746- 4436
FAX: 212-746-8167
EM: sandjel@med.cornell.edu

Zoran Popmihajlov, M.D.
Weill Medical College
Cornell University
Division of Immunology
Department of Medicine
515 E. 71st St.
New York, NY 10021
Phone: 212-746-4406
EM: zop2001@med.cornell.edu

Nurse Practitioner

Liza Kelly-Rossini, M.P.H., A.N.P.
Weill Medical College
Cornell University
Division of Immunology
Department of Medicine
515 East 71st St.
New York, NY 10021
Phone: 212-746-4435
FAX: 212-746-8167
EM: lik2009@med.cornell.edu

Clinical Trials Coordinator

Aquanette T. Sass
Weill Medical College
Cornell University
Division of Immunology
Department of Medicine
515 East 71st St.
New York, NY 10021
Phone: 212-746-4361
FAX: 212-746-8167
EM: aqs2001@med.cornell.edu

Laboratory Technician

Huili Ma
Weill Medical College
Cornell University
Division of Immunology
Department of Medicine
515 East 71st St.
New York, NY 10021
Phone: 212-746-4406
EM:
hum2001@med.cornell.edu

Statistician

Martin Lesser, Ph.D.
Director, Biostatistics Unit
General Clinical Research Center
Weill Medical College
Cornell University
1300 York Avenue
New York, NY 100212
Phone: 516-240-8300
FAX: 516-240-8344
EM: marty@nshs.edu

Protocol Pharmacist

Steven Benkert, R.Ph.
Investigational Drug Service
New York Presbyterian Hospital
525 East 68th St.
New York, NY 10021
Phone: 212-746-0743
FAX: 212-746-0789
EM: ftb9010@nyp.org

Data Manager

Elizabeth Wood
Weill Medical College
Cornell University
Director, Data Management Unit
General Clinical Research Center
1300 York Avenue
New York, NY 10021
Phone: 212-746-4744
EM: eawood@med.cornell.edu

Aventis Pasteur Sponsor

Raphaelle El Habib, Ph.D.
Chef de Project Vaccin VIH
Aventis Pasteur SA
Campus Merieux
1541, Avenue Marcel Merieux
Marcy L'Etoile F-69280, France
Phone: 33-4-37-37-32-28
EM: Raphaelle.elhabib@aventis.com

General Clinical Research Center
Outpatient Staff

Sandra Flynn, RN
Mark Vieux - Phlebotomist
Carol Brooke, Secretary-Receptionist
Weill Medical College
Cornell University
General Clinical Research Center
1300 York Avenue
New York, NY 10021
Phone: 212-746-4762Becton Dickinson
Sponsor

Cory Waters, Ph.D.
Medical Affairs
BD Biosciences
2350 Qume Dr.
San Jose, CA 95131-1807
Phone: 408-954-6314
EM: Cory_Waters@bdis.com

Chiron Sponsor

Anne-Marie Duliege, M.D.
Senior Medical Director
Clinical Research & Development
Chiron Corp.
4560 Horton St.
Emeryville, CA 94608-2916
Phone: 510-923-2715
EM: anne-marie_duliege@Chiron.com

2 STUDY MANAGEMENT

This is a single center study conducted by the Weill Medical College of Cornell University. The administrative offices are housed at 515 East 71st St., S-208 (Dr. Smith, PI & Protocol Chair) and S-212 (Ms. Liza Kelly-Rossini, Clinical Trials Coordinator; Nurse Practitioner). The Study Team will meet weekly to review the study management.. The study will be conducted at the Weill Cornell General Clinical Research Center, which is located at 525 East 68th St., Payson 2, and at 99 University Place.

For clinical medical management, including entry criteria, adverse event management, concomitant medications, and co-enrollment, the Protocol Chair will respond.

For questions specifically related to laboratory tests performed by the New York Presbyterian Hospital (NYPH) Laboratories, the Clinical Trials Coordinator will respond. For questions related to immunologic assays performed in the Immunology Division, the Division Laboratory Technologist will respond.

For non-clinical questions about inclusion/exclusion criteria, the schedule of events, database entry, randomization/registration, transfers, delinquencies, and other data management issues, the Clinical Trials Coordinator will respond.

For protocol questions, and copies of the protocol, the Clinical Trials Coordinator will respond.

For questions or problems relating to study drugs, dose, supplies and returns, the Protocol Pharmacist will respond.

3 SCHEMA

DESIGN

This is a two-step, phase II, randomized, partially double blinded, single center 2X2 factorial study to determine the efficacy of immunotherapeutic approaches to control or prevent resumption of detectable plasma HIV concentrations upon interruption of Highly Active Anti-Retroviral therapy (HAART). Two immunotherapeutic approaches will be evaluated; therapeutic HIV vaccination (vCP1452) and daily low-dose interleukin 2 (IL2) administration. The efficacy of these immunological therapies will be determined by monitoring the dynamics of viral rebound upon cessation of antiviral therapy. Prior to enrollment, plasma HIV RNA concentration must be undetectable and the circulating CD4+ T cell concentration must be > 400 cells/mm^3. Subjects must not have undergone virological failure (i.e. > 10,000 HIV RNA molecules/ml) while receiving HAART.

DURATION: Subjects will be on study for 36 weeks.

SAMPLE SIZE: 92 subjects will be randomized into 4 arms of 23 subjects each.

POPULATION: Adults with chronic HIV infection with entry CD4+ counts $\geq$ 400 cells/mm^3, who have achieved suppression of plasma viremia while on HAART.

SCHEDULE: The schedule of events will be divided into Step I (HAART and immunotherapeutic interventions: weeks 0-12), and Step II (interruption of HAART: weeks 13-36), Those individuals randomized to receive IL2 in Step I will continue to receive IL2 in Step II. Step II will be extended for an additional 12 weeks for those individuals who meet the criteria to remain on study at the end of Step II, i.e. (VL <30,000 and CD4+ counts >250 cells/mm^3 and/or > 50% of baseline CD4+ counts.) and will be referred to as Step III. During this Step III phase, individuals will continue off HAART and on IL-2 if applicable.

STEP I: In addition to continuing to receive HAART, subjects will be randomized into one of the following four arms:

A. Immunization placebo;
B. Immunization with the canarypox HIV-vaccine (vCP1452);
C. Daily low-dose IL-2 + immunization placebo;
D. Daily low-dose IL-2 + canarypox HIV-vaccine (vCP1452).

Subjects on Arms A, B, C, or D will receive vaccine (or vaccine placebo) injections at weeks 0, 4, 8, and 12. For subjects on Arms C or D, IL-2 will be administered by daily subcutaneous self-injection. HAART will not be provided as part of this study

STEP II: Subjects on all arms (A, B, C, D) who meet inclusion criteria will proceed to Step II, and interrupt HAART for a minimum of 12 weeks and a maximum of 24 weeks.

STEP III: Subjects on all arms (A, B, C, D) at the end of 12 weeks of Step II whose viral load remains < 30,000 copies/mL and continue to remain off HAART and on IL-2 (if applicable) for an additional 12 weeks. Subjects will not terminate Step III unless and until their viral load increases to >30,000 and/or CD4 count decreases to <250 cells/mm^3 or 50% of the baseline CD4+ T cell concentration on 2 successive occasions. Monitoring of viral loads and T cell counts will continue bi-weekly, providing a total of 36 weeks on study. At the end of week 36 subjects are deemed off study and will discontinue IL-2 if applicable. A close out visit shall be conducted within 2 weeks of completing Step III.

4 OBJECTIVE:

To determine whether HIV-specific canarypox vector immunizations and/or daily low dose IL2 result in efficient immune control of viral replication subsequent to an interruption of HAART.

4.1 Primary Endpoints

To compare the proportion of subjects among the 4 groups of subjects who relapse during the first 12 weeks following cessation of HAART.

To compare the mean $\log_{10}$ viral load for each experimental group from the average of 5 values obtained during study weeks 21-25, which corresponds to weeks 8-12 following the interruption of HAART.

To compare the proportion of subjects among the 4 groups who are eligible to progress to step III.

4.2 Secondary Endpoints:

- To compare the concentrations of circulating CD4+ and CD8+ T cells among the 4 experimental groups.
- To compare changes in the frequency, activation state, and HIV-specific functional capacity of T cells and NK cells in blood as monitored by the expression of intracellular cytokines among the 4 groups of subjects as assessed by flow cytometry during the first 12 weeks after the cessation of

HAART, and in relationship to the time interval to the termination of Step II.

5 INTRODUCTION

5.1 Background & Rationale:

HAART is effective in suppressing replication of HIV [1,2], but it is now known that it cannot cure the infection. Even after several years of viral suppression to undetectable plasma levels, upon cessation of therapy, all patients reported thus far have suffered a viral relapse within a few days to a few weeks[3, 9]. Accordingly, most practitioners now realize that it will be very difficult, if not impossible, to completely eliminate or eradicate the microbe. In this regard, acute viral infections are combated and eventually controlled by the development of cell-mediated immunity[10, 14]. However, in the case of HIV, it has become accepted as dogma that individuals chronically infected with HIV have suffered irreparable damage to the immune system as a consequence of viral targeting and destruction of CD4+ T cells, which prevents elimination of the virus and the development of protective immunity[15].

The generation of a maximal cellular immune response to acute viral infections is dependent upon a rapid and marked proliferation of lymphocytes, especially of the cytotoxic T cell subset, which express CD8 surface molecules. This proliferation of CD8+ T cells is dependent upon the CD4+ T cell subset, which "helps" CD8+ T cells by producing large amounts of the T cell growth factor, IL2 [16, 17]. If either CD4+ T cells or IL2 are limited, then expansion of CD8+ T cells is truncated [18]. Because CD8+ T cells react with virally infected cells by secreting antiviral cytokines such as interferon-γ (IFN-γ) and tumor necrosis factor-α (TNF-α), and by direct cell-cell killing via the secretion of cytolytic molecules, such as Perforin and Granzymes, the absolute number of antigen-reactive CD8+ T cells largely dictates the success of the cellular immune reaction [12, 19, 20].

To investigate whether individuals who are infected chronically with HIV indeed could mount immune response to HIV, we have been conducting clinical trials, testing the effect of IL2 therapy on the host capacity to regulate plasma HIV concentration upon interruption of HAART. We initiated a protocol to interrupt HAART when individuals had recovered normal levels of circulating lymphocytes and had undetectable plasma HIV concentrations [21]. Thus far, we have studied 15 subjects and all individuals have suffered a viral relapse when HAART has been interrupted. The time to detectable viremia has been 19 ± 3 days. Once detectable, the plasma HIV concentration increased rapidly with a doubling time of 1.6 ± 0.3 days, reaching a peak concentration within 2 weeks. Thereafter, the plasma HIV concentration declined with a half time (t ½) of 3.5 ± 0.5 days, ultimately reaching a trough concentration within 6-8 weeks from the time of onset of detectable viremia. Data from the first 9 subjects revealed that the trough HIV concentration = 4.2 ± 0.5 (1 SD) $\log_{10}$ HIV RNA molecules/ml. Based on this experience, we have elected a minimum of 12 weeks post the cessation of HAART to determine the viral dynamics for this protocol.

Coincident with the rise and subsequent decline of the plasma HIV concentration was an increase in the concentration of circulating CD8+ T cells, such that soon after the peak viral titer, the CD8+ T cell concentration doubled from baseline. Moreover, the rate and magnitude of the decline in HIV concentration after the peak correlated with the magnitude of increase in the circulating CD8+ T cells. Therefore, these data suggested that chronically infected individuals ***are*** capable of mounting a significant antiviral host response, which correlates with an increase in the CTL compartment. By comparison, the circulating CD4+ T cells underwent only a transient decrease from baseline, ~ 25%, while the concentration of NK cells remained unchanged.

Subsequently, several individuals remained off of HAART but continued to take IL2, some for periods as long as 10 months, and with plasma HIV concentrations < 10,000 molecules/ml. To test whether any of these subjects had developed anamnesis as a consequence of the first interruption of HAART, 4 subjects resumed HAART until the plasma viral concentration once again became undetectable, and then discontinued the HAART for the second time. These subjects all suffered a second viral relapse, with a similar latent time to the onset of viremia, and a similar doubling time and time to the peak of plasma virus concentration, ~ 14 days, compared with the first cessation of HAART. However, the peak and trough plasma HIV concentrations were >10-fold lower after the second cessation in 3 of the 4 subjects.

These data support the interpretation that individuals infected chronically with HIV before antiretroviral therapy is initiated retain sufficient recognition of HIV antigens to mount a detectable antiviral host response once the antivirals are withdrawn. However, the level of the response is not sufficient to prevent viral relapse. The correlation of the rate and magnitude of the viral decline with the magnitude of the increment in CD8+T cell concentration suggests that the CD8+ T cell arm of the host cellular immune system is responsible, at least in part, for the antiviral effect. However, by analogy to other viral infections, the CD4+ T cell arm of the response is probably also involved. In addition, the antiviral activity of NK cells may also play a role in the results that we have obtained thus far, in that daily low dose IL2 therapy elevates the concentration of circulating NK cells [22]. All of these data provide the rationale for further investigation of immunotherapeutic approaches designed to augment HIV-specific immune reactivity in the treatment of HIV+ individuals.

The viral dynamics of the relapse provide the data necessary to plan the next immunotherapeutic trial. Accordingly, we plan to perform the immunotherapeutic manipulations while the endogenous viral load is maximally suppressed by HAART. The endpoint of the study will be determined using the viral dynamics of the relapse that occurs after cessation of HAART. We plan to follow subjects for a minimum of 12 weeks after treatment interruption and carefully document and compare the plasma HIV RNA concentrations among the different study groups. Secondary viral endpoints will be the proportion of individuals who relapse within

this time period, and as well, the time interval between the cessation of HAART and the reinstitution of HAART.

The lymphocyte dynamics that we have observed will serve as additional secondary endpoints. Thus, the rate and magnitude of changes in CD8+ T cell concentrations, as well as any changes in CD4+ T cells and NK cells will be monitored weekly during the first 12 weeks following the cessation of HAART. In addition to any changes in circulating lymphocyte subsets, we will also determine the state of cellular activation of the cells before and after a short-term activation *in vitro* with HIV antigens and peptides (23-26).

Because there was not a detectable increase in circulating CD8+ T cells until after the return of detectable plasma HIV RNA, it appears that viral replication is not prevented from re-initiation, but there is a threshold of antigen concentration that is necessary to mobilize the host response. Also, based upon detailed lymphocyte dynamics after the suppression of viral replication by HAART, an increment of lymphocytes does not appear in the circulation until there is a marked diminution of viral replication in the secondary lymphoid tissues 23]. Therefore, we are operating from the hypothesis that an effective antiviral immune response requires a combination of antigenic stimulation and IL2-induced proliferation and differentiation. This concept is also supported by our detailed studies of the regulation of the T cell immune response, which indicate that the expression of IL2 receptors is tightly controlled by T cell antigen receptor stimulation, and is transient after antigen activation ([24, 27]).

5.2 Canarypox vaccines (vCP1452, ALVAC)

While HIV-specific immunization of normal, HIV- individuals has been safely carried out in a number of clinical trials[28, 30], there is still limited information regarding the administration of canarypox vectors to HIV-infected individuals. No toxicity was observed in the initial conventional laboratory testing of live vector vaccine constructs including vaccinia and canarypox agents[31]. Because of the theoretical potential for vaccinia vectors to replicate within immunosuppressed individuals, there was a desire to develop other vaccine approaches targeting CTL responses without the same safety concerns. In contrast to vaccinia virus, canarypox does not produce infectious progeny in mammalian hosts[32]There has been no evidence of canarypox replication thus far in human hosts, and in preliminary testing there have been no significant adverse events when canarypox agents were administered to immunosuppressed animal models or HIV-infected adults[28]

ALVAC (2) 120 (B, MN) GNP (vCP1452) is a preparation of a modified recombinant canarypox virus, ALVAC), expressing the gene products of the HIV-1 *env* and *gag* genes, and a synthetic polypeptide encompassing the known human CTL epitopes from the *nef* and *pol* gene products. ALVAC is a plaque-purified isolate of an attenuated canarypox virus into which the vaccinia virus E3L and K3L

coding sequences are inserted. Virogenetics Corporation, Troy, New York, developed the recombinant vaccine construct. Aventis Pasteur, Marcy L´Etoile, France manufactures this experimental vaccine.

The final product is diluted with freeze drying medium (amino acids of BME medium, Tris 10mM, and sodium glutamate), poured into vials and lyophilized. The lyophilized vaccine is reconstituted with sterile isotonic solution (NaCl 0.4%) supplied with the package.

5.2.1 Pre-clinical Studies:

Recombinant canarypox virus does not produce infectious progeny virus in mammalian cells. Only Vero cells were shown to allow low growth of one specific ALVAC construct. However, no replication of ALVAC vCP1452 could be detected in vitro on numerous human cell lines and Vero cells and in vivo in human volunteers. In tissue culture tests performed with ALVAC (i.e. ALVAC-RG [ALVAC vector expressing the Rabies G Glycoprotein]) to ascertain whether or not the recombinant could be adapted to grow in non-avian cells, results indicate that adaptation could not be demonstrated [33].

Therefore, while the HIV-1 *env*, *gag*, protease genes, and the nef/pol CTL polypeptide encoded in the canarypox recombinant, ALVAC (14) HIV (vCP1452), are expressed in mammalian hosts, canarypox virus replication is not established. The risk of transmission and progressive disease is consequently eliminated[34, 35].

5.2.1.1 ALVAC-HIV (vCP1452)-infected autologous PBMCs stimulate CTLs in vitro

PBMC samples derived from HIV-seropositive individuals were inoculated with ALVAC-HIV recombinants according to the methods of Ferrari et al. 1997 [36] and used to stimulate HIV-specific cytolytic activity from autologous PBMCs. The results demonstrate the ability of ALVAC (14) HIV (vCP1452)-infected cells to stimulate HIV-1 Env-, Gag-, Nef-, and Pol-specific cytolytic activity in vitro.

These results confirm expression of the HIV-1 Env, Gag, Nef and Pol components in ALVAC (14) HIV (vCP1452)-infected cells and the presentation of HIV-1 Env-, Gag-Nef-, and Pol-specific peptides corresponding to human CTL epitopes in the context of MHC class I molecules.

5.2.1.2 Safety of canarypox

An avian poxvirus vector (canarypox) is being used to express genes encoding HIV-1 antigens as well as other vaccine antigens, such as rabies glycoprotein and measles hemagglutinin and fusion proteins. In principle, the canarypox-vectored vaccine (ALVAC) that expresses one or more antigens of HIV-1 might satisfy many of the criteria for an affordable HIV vaccine. Like vaccinia virus, canarypox

can accommodate large amounts of foreign DNA in its genome, infect mammalian cells and cause them to produce foreign proteins. It is thermostable. In contrast to vaccinia virus, canarypox virus is host-range restricted. In mammalian cells, it undergoes an abortive cycle of replication and does not produce infectious progeny virus[32, 35]. High doses of canarypox virus have not caused adverse effects in a wide variety of animals, even profoundly immunosuppressed animals. This suggests that canarypox recombinants are not likely to disseminate and cause progressive disease in human recipients or be transmitted to unvaccinated contacts. Canarypox vectors have been used in clinical trials of rabies, measles, a Japanese encephalitis virus, and CMV vaccines and vaccines containing the HIV envelope or other genes. Recombinant canarypox rabies glycoprotein is well tolerated and immunogenic. The immune response is dose dependent; after two immunizations, approximately 75% developed serum antibodies against rabies [34]

5.2.2 Safety in HIV Seronegative Subjects

ALVAC candidate vaccines expressing proteins from rabies, measles, CMV, Japanese encephalitis, or HIV have been administered to a total of 1,364 HIV seronegative individuals (data from Aventis Pasteur). No severe (Grade III or Grade IV) reactions have been observed that were attributable to the vaccine.

In a safety and immunogenicity trial involving the administration of a canarypox vector expressing gp120 (ALVAC) with or without subunit gp120 boosts to HIV-uninfected adults, the immunizations were well tolerated[28]. All adverse events were self-limited. The most common adverse events reported were mild to moderate local pain or tenderness at the vaccine site (70-90%). Approximately 10% of subjects receiving ALVAC or ALVAC+gp120 reported a severe symptom at least once during a series of immunizations. These included large erythematous vaccine site reactions, occasional headaches, and one report each of vomiting and severe malaise. These events were not consistently seen throughout the study, in that, for example, subjects who experienced an episode of headache or erythematous reaction did not typically suffer a recurrence with subsequent injections. A single subject who received ALVAC + gp120 had an apparent decline in absolute CD4 count from 595 at baseline to 280 after the 4th injection, but a repeat CD4 count two weeks later was 481. None of the other subjects (n=120) receiving ALVAC constructs had significant changes in clinical laboratory results, including CD4 and CD8 counts.

5.2.3 Carcinogenesis, Mutagenesis, Impairment of Fertility:

This vaccine has not been evaluated for its carcinogenic, mutagenic potential, or impairment of fertility.

5.2.4 Pregnancy:

Animal reproductive studies have not been conducted with this vaccine. It is also not known whether this vaccine can cause fetal harm when administered to a pregnant woman or can affect a woman's reproductive capacity. This vaccine should not be given to pregnant women, and all women of reproductive potential must use an acceptable form of birth control during clinical trial involvement.

5.2.5 ALVAC-HIV vCP1452 - to be studied in this protocol

The clinical experience with ALVAC-HIV (vCP1452) in humans is less extensive. However, by August 2000, the product has been administered to 100 HIV negative volunteers (35 in the AVEG 034 study, 40 in the AVEG 034A study, 3 in the LIP03 study, and 22 in the ongoing ANRS VAC 010 study), and 12 HIV infected patients in the ongoing ADARC01 study, as well as 14 in the ACTG 5058 study. No serious adverse reaction related to the vaccine was reported in any of these trials.

ALVAC-HIV (vCP1452) is well tolerated. For example, in the AVEG 034 study, 134 injections were administered to 17 men and 18 women. Systemic reactions of any grade were rare, and there were no severe, Grade III or IV systemic reactions. Moderate reactions were malaise in 4 patients, myalgia in 6, headache in 5 and fever in 1 patient. Severe local reactions were rare, with tenderness, erythema >25 cm2 and induration in one patient, whereas moderate reactions were more frequent, with pain and/or tenderness in 24 patients.

Most of HIV-infected patients immunized with ALVAC-HIV (vCP1452) experienced soreness at the injection site for a short time, but there were no systemic or serious reactions. ALVAC-HIV (vCP1452) did not result in a rebound in the HIV viral load as measured by RT-PCR within 1-51 days post injection.

5.2.5.1 Safety record of the ALVAC product in HIV infection

According to data provided by the manufacturer, ALVAC candidate vaccines have been administered to at least 26 HIV-infected individuals to date. In study of vCP1452 in HIV-infected individuals at the Aaron Diamond AIDS Research Institute, 14subjects have been immunized to date [37]. Most experienced soreness at the injection site for a short time (hours), but there were no systemic or serious local reactions. ALVAC-HIV (vCP1452) did not result in a rebound in the HIV viral load as measured by RT-PCR within 1 to 51 days post injection. In addition, ALVAC vCP125 was administered to 10 HIV-positive subjects with no severe (Grade 3 or Grade 4) reactions. As a control vaccine, ALVAC-rabies was given to 10 HIV-positive subjects with no severe reactions (Dr. C. Katlama via Aventis Pasteur).

5.2.5.2 Immunogenicity of previous ALVAC-HIV vectors

Previous canary pox vectors expressing HIV proteins have induced CTL in approximately 40% of HIV-uninfected subjects on at least one occasion, and in approximately 25% on 2 or more measurements. We postulate that in HIV-infected subjects in whom HIV replication has been suppressed with potent antiretroviral therapy that vCP1452 will express sufficient HIV epitopes to boost CTL activity to detectable levels in a majority of subjects. Moreover, a combination of HIV vaccine and daily low-dose IL2 administration should be more effective in augmenting HIV-specific immune reactivity than either alone. . In a recent study from The Aaron Diamond AIDS Research Center, of 14 subjects who received 4 injections of vCP1452, 13 (93%) had significant increases in anti-gp160 or anti-p24 antibody titers, and 9/14 (64%) had transient augmentation of their T cell proliferative responses to gp160 and/or p24 HIV antigens. CD8+ T cell responses to vaccinia virus expressing HIV antigens (*env, gag, pol, nef, ctrl)* assayed via flow cytometry of IFN-γ-producing cells, yielded positive responses in 11 subjects (79%), and 7 subjects had responses to more than 1 antigen[37].

5.3 Daily Low-Dose IL2 Administration to HIV+ Individuals

5.3.1 Phase I

In 1994 we initiated a phase I dose-finding/safety study to determine a dose of IL2 that could be administered safely (i.e. would not result in the stimulation of viral replication), and without any systemic side effects, to chronically infected individuals. The Amgen Corporation manufactured the preparation of IL2. We found the maximum non-toxic dose to be 250,000 Units/M^2, given as a daily subcutaneous injection[22]. With a specific activity of 15,000,000 U/mg, this amounted to 16.7 μg, which in a normal adult with a BSA of 1.5 M^2, amounts to ~ 25 μg/day. This dose was administered for 24 weeks to 10 individuals who also received monotherapy with a nucleoside analogue. There were no constitutional symptoms noted during this interval, and no significant change in plasma virus concentration from the baseline determinations. However, there were readily detectable and significant changes in immunologic parameters, including increments in circulating NK cells, eosinophils and monocytes. Pertinent to the CD4+ T cell deficiency of HIV infection, there was a progressive increase in circulating CD4+ T cells. In addition, there was a significant increase in the frequency and magnitude of delayed-type hypersensitivity reactions to common environmental antigens[22]

5.3.2 Phase I/II

On the basis of these findings, in 1996 we initiated a phase I/II uncontrolled study to test this dose and regimen of Amgen IL2 in a larger number of individuals. Forty individuals with circulating CD4+ T cells between 200-500 cells/ml were treated

with HAART and daily low-dose IL2 for 48 weeks. The results confirmed and extended our earlier findings[38], in that there was no systemic toxicity, and there were readily detectable enhancement of the concentrations of circulating CD4+ T cells, at a rate of 10-cells/μl/month, which continued for the entire 12-month interval. This rate of increase is ~ 2.5-fold more rapid than reported for similar cohorts of individuals who were treated with HAART alone. In addition, the concentration of NK cells increased at a rate of 80-cells/μl/month for the first 3 months, then remained elevated on a plateau for the next 9 months. Since the average NK cell concentration at baseline was ~ 100 cells/μl, this resulted in an increment of ~ 3-4-fold above baseline. As noted previously, there was a rapid increase in circulating eosinophils, with a maximum of ~ 8-10-fold increase apparent already after the initial 14 days of therapy. Of particular significance, there were no untoward systemic toxicities noted in this longer-term study[38]

5.3.3 Amgen IL2 vs. Chiron IL2

Upon completion of the phase I/II study, the Amgen Corporation discontinued the manufacture of IL2. Therefore, we performed a series of *in vitro* IL2 bioassays and ELISAs to determine whether the Amgen IL2 preparation and IL2 manufactured by the Chiron Corporation (Proleukin) were comparable. We found that the Amgen preparation was ~ 5-fold more potent than the Chiron IL2 preparation. Thus, 250,000 U (16.7 μg) of Amgen IL2 is equivalent to 1.25 million U (133 μg) of Proleukin. Pharmacokinetic studies in HIV+ individuals comparing the Amgen IL2 preparation with Proleukin confirmed these *in vitro* findings. Only when ~ 5-fold greater amounts of Proleukin were administered, compared with the Amgen preparation, were equivalent plasma IL2 concentrations attained.

5.3.4 Phase II

On the basis of these findings, the Chiron Corporation sponsored a phase II randomized, multi-center controlled trial to test Proleukin in chronically infected individuals who were identified to have <300 circulating CD4+ T cells/μL while receiving an effective HAART regimen. The Weill Medical college of Cornell University served as the institution of the Principle Investigators, and 11 institutions participated. Although this study did not exactly repeat our previous phase I/II study in individuals who had 200-500 circulating CD4+ T cell/μL, it was felt that this study would benefit those individuals most in need of immune enhancement

The results of the randomization of 108 subjects confirmed and extended our earlier findings, in that there was a rapid and significant increment in circulating NK cells over the first 3 months of the 6-month trial, and a gradual and progressive increase in CD4+ T cells in the IL2 treatment arm[39]. Of particular importance, there was a significant increase in CD4+ T cells with a naïve phenotype (i.e. CD45 RA), compared with a memory phenotype (CD45 RO). As well, the number of serious, Grade III and IV adverse events encountered with daily, low dose IL2 was significantly lower than observed in previous trials when ~10-fold higher doses (i.e.

15 mU/day) of Chiron IL2 were administered[40]

5.3.5 IL2 Studies to Augment Antigen-Specific Immunity

On the basis of the studies completed to date, it has become established that the daily administration of IL2 to HIV+ individuals for intervals of ≥ 24 weeks is both safe and nontoxic whether or not effective viral suppressive medications are also administered [38, 39]. Accordingly, the administration of IL2 to supplement any endogenous IL2 produced during the resumption of viral replication upon cessation of HAART would be expected to yield no untoward events, and as well, should be supportive of maximal immune responses to viral antigens, especially if the production of endogenous IL2 is deficient. Our experience to date is consistent with this interpretation. As detailed in the Background Section, after treatment interruption and continuation of IL2 therapy there is a CD8+ lymphocytosis that is dependent temporally upon detectable viremia. This CD8+ lymphocytosis has not been reported following treatment interruption in the absence of daily IL2 therapy[9]. Since CD8+ T cell proliferation is IL2-dependent, we hypothesize that the responses observed thus far are ascribable to the IL2 therapy. However, this assumption must be tested in a controlled fashion, as intended by this study.

6 STUDY DESIGN

This is a phase II, randomized, controlled, partially doubly blinded, single center, 2X2 factorial two-step study to test whether immune-based therapies consisting of an HIV canarypox vaccine and or daily low dose IL2 administration provides augmented host antiviral activity as tested by monitoring plasma HIV concentration upon cessation of Highly Active Anti-Retroviral Therapy (HAART). Volunteers will be randomized to one of 4 groups in Step I as follows:

1) Vaccine placebo
2) Vaccine
3) Vaccine Placebo + IL2
4) Vaccine + IL2

Subjects will receive 4 injections of vaccine/placebo at weeks 0, 4, 8, and 12. Those subjects randomized to receive IL2 will self-administer daily IL2 injections throughout the entire Step I interval of 12 weeks.

At the end of Step I, subjects who meet eligibility criteria will enter Step II, when HAART is discontinued for a minimum of 12 weeks. At the end of Step II, subjects will be declared off protocol if the plasma HIV concentration ≥ 30,000 HIV RNA mol/mL or the circulating CD4+ T cell concentration falls below 250 cells/µL or ≤ 50% of the mean baseline CD4+ T cell concentration. If these thresholds are not met in the last 2 determinations, the subjects will remain on study and enter Step III, on protocol for another 12 weeks. During this interval, if these HIV and CD4+ T cell thresholds are surpassed on two successive determinations the subject will be

declared off protocol. At the end of the Step III, all subjects will be declared off protocol and will discontinue IL-2. If a subject has completed Step II but does not meet criteria to continue on Protocol in Step III, they are recommended to restart HAART.

When a subject has completed the protocol, decisions regarding future HIV medications will revert to the subject's health care provider.

7 SELECTION AND ENROLLMENT OF SUBJECTS

7.1 Step I: Receipt of Study Medication(s) and Potent Antiretroviral Therapy

7.1.1 Inclusion Criteria: Step I

1. Documentation of HIV seropositivity by ELISA and Western Blot analysis
2. Male and female subjects ≥ 18 years ≤ 65 years
3. Ability and willingness to sign an informed consent.
4. Receiving stable HAART, defined as 2 or more antiretroviral drugs in combination. Changes in drugs are allowed if due to any reason other than virologic failure.
5. Documentation of CD4+ T cell concentrations never < 200 cells/µL within the 12 months prior to enrollment, and CD4+ T cell concentrations of ≥ 400 cells/µL on two successive occasions at least 14 days apart within 30 days of study entry.
6. Documentation of plasma HIV RNA concentrations never > 2 million molecules/ml, and suppression on HAART to < 50 molecules/ml on 2 successive occasions at least 14 days apart within 30 days of entry.
7. No history of virologic failure (i.e. >10,000 HIV RNA molecules/ml) while receiving current HAART regimen.
8. Women of reproductive potential (defined for this study as sexually mature women who have not been post-menopausal for at least 24 consecutive months, or have not undergone hysterectomy or oophorectomy) must have a negative serum or urine pregnancy test with a sensitivity of at least 50 MIU/mL performed within 30 days prior to enrollment and again within 24 hours before initiating study therapy.

   All subjects must not participate in a conception process, and if participating in sexual activity that could lead to pregnancy, male subjects must take every precaution to avoid risk of pregnancy for their female partners, women subjects/male partners must use two reliable methods of contraception simultaneously, while receiving study therapy and for 6 months following permanent discontinuation of study therapy.

   NOTE A: Reliable forms of contraception are a combination of two of the following methods: 1) condoms (male or female) with or without a

spermicidal agent. 2) Diaphragm or cervical cap with spermicidal, 3) IUD, or 4) hormonal-based contraception.

NOTE B: An IUD is an adequate method of contraception but increases the risk of pelvic inflammatory disease.

7.1.2 Exclusion Criteria: Step I

1. A current AIDS-defining illness.
2. Documentation of virologic failure (i.e. >10,000 HIV RNA molecules/ml) while receiving current HAART regimen.
3. Laboratory tests within 30 days of entry as follows:
    a. Hgb <9.1 g/dL for males & <8.9 g/dL for females
    b. ANC < 750 cells/µL
    c. Platelet < 75,000 cells/µL
    d. AST > 3 x ULN
    e. ALT >3 x ULN
    f. Serum creatinine > 1.5 x ULN
    g. Total bilirubin ≥1.5 x ULN
4. History of immunomodulating agents including: other interleukins (not including IL-2), antibodies reactive with lymphocytes, monocytes or antigen presenting cells, and polyribonucleotides.
5. Uncontrolled active cardiac, renal, pulmonary, hepatic or CNS disease.
6. IL2 therapy within 4 weeks of entry.

7. History of active malignancy requiring chemotherapy.
8. History of thyroid disease, autoimmune disorders including asthma, inflammatory bowel disease, rheumatoid arthritis and psoriasis.
9. Active substance abuse that will compromise the subject’s ability to adhere to the study requirements.
10. Alcohol dependency, for example, current alcohol use of more than 1 drink/day any time during the previous 6 months. One drink is defined as 12 ounces of beer, 5 ounces of wine, or 1-¼ ounces of hard liquor.

11. History of allergy to eggs, or to IL2, or other components of the vaccine or IL2 formulation, i.e. phosphate buffer, SDS, mannitol. RBV: cellulose, lactose, croscarmellose, magnesium stearate, Blue pharmaceutical ink: shellac, anhydrous ethyl alcohol, isopropyl alcohol, n-butyl alcohol, propylene glycol, ammonium hydroxide, Blue # 2 aluminum lake.
12. Active co-infection with Hepatitis B Virus or Hepatitis C Virus, as defined by detectable viremia.
13. . Pregnant or breast-feeding women
14. Professionals working in close contact with canaries (e.g., breeding farms, bird shops), who are likely to have antibodies to canarypox prior to vaccination.

15. Severe retinopathy due to diabetes, hypertension, CMV, or macular degeneration
16. Acute therapy for a serious infection or other serious medical illness that is potentially life threatening and requires systemic therapy and/or hospitalization within 14 days of study entry.
17. Serum creatinine > 2.0 mg/dL or creatinine clearance < 50 mL/min as estimated by the Cockcroft-Gault equation. This screening value must be obtained within 30 days of enrollment.
18 History of major organ transplantation with an existing graft.
19.Subject judged by the investigator to be at significant risk of failing to comply with the provisions of the protocol as to cause harm to self or seriously interfere with the validity of the study results.

7.1.3 Inclusion of women and minorities:

The volunteers for this study will be adult residents of the greater metropolitan New York area. As such we anticipate that they will be racially disparate. Because HIV infection has become manifest predominantly in homosexual males, we anticipate that the gender distribution will be skewed towards males. Because there is a large Hispanic population in NYC, we anticipate that a greater proportion of both male and female Hispanic people will volunteer. Pregnant women, children, prisoners and institutionalized individuals will be excluded from this study.

TABLE I: NATIONAL DEMOGRAPHICS FOR DISEASE/DISORDER (percent):

	**American Indian or Alaskan Native %**	**Asian or Pacific Islander %**	**Black, not of Hispanic Origin %**	**Hispanic %**	**White, not of Hispanic Origin %**	**Other or Unknown %**	**Total %**
Female	0-1	0-1	10	3	4	0	18
Male	0-1	1	28	13	40	0	82
Unknown							
Total	0-1	1-2	38	16	44	0	100

REFERENCE FOR DEMOGRAPHICS: **www.CDC.gov/hiv/stats/cumulati.htm**

Table II: FINAL SUBJECT POPULATION TO BE RECRUITED (Numbers): The total number should equal the final number of subjects to be recruited. The numbers in this table should reflect the anticipated result of the plan for gender and minority recruitment.

	**American Indian or Alaskan Native (N)**	**Asian or Pacific Islander (N)**	**Black, not of Hispanic Origin (N)**	**Hispanic (N)**	**White, not of Hispanic Origin (N)**	**Other or Unknown (N)**	**Total (N)**
Female	0	0	9	3	5	0	17
Male	0	1	26	12	36	0	75
Unknown							
Total	0	1	35	15	41	0	92

7.1.4 Recruitment

Volunteers will be recruited primarily through contact with their physicians. We plan to inform physicians who have large practices dealing with HIV infections via phone and mailings. We have established a network of physicians who have referred patients to our previous studies, and we also have a close working relationship with 4 HIV treatment centers within the city. We plan to place IRB-approved advertisements in various strategic clinics within the city, to appeal directly to patients and conduct patient education forums. As well, we will register our protocol with various clinical trials communications sites, post advertisements in newspapers and magazines and develop print campaigns.

7.1.5 **Criteria for withdrawal from Step I: Inability to maintain viral control:**

The subject must be withdrawn from the study if the Viral load ≥ 500 HIV RNA mol/mL on two successive determinations at any time during Step I. If the HIV RNA is > 50 HIV RNA mol/ mL at week 12, this value must be repeated. If still >50 HIV RNA mol/mL, the subject will not be eligible for continuation onto step II and must be withdrawn from the study.

7.2 Step II: Interruption of Antiretroviral Therapy:

7.2.1 Inclusion Criteria

1. Completion of a minimum of 12 weeks on Step I.
2. Undetectable plasma HIV RNA concentration on week 12.
3. The mean CD4+ T cell concentration during Step I $\geq$ 350 cells/uL, or at least 70% of baseline concentration for each subject.
4. Receipt of 4 doses of either vaccine or vaccine placebo (weeks 0, 4, 8, and 12).
5. Those subjects randomized to the groups that receive IL2 must have received at least 12 weeks of IL2.

7.2.2 Exclusion Criteria

1. Laboratory values that exceed the ranges listed as exclusionary for entrance to Step I. If an isolated laboratory value falls beyond these ranges, it may be repeated, and if within the ranges then the subject may proceed to Step II.
2. Development of an intercurrent illness, which in the opinion of the investigator and/or PMD would contraindicate the discontinuation of HAART.
3. Non-adherence with study visits or medications during Step I, which in the opinion of the investigator would interfere with the subject's ability to complete the trial.

7.3 Criteria to terminate Step II

1. At week 25, Step II will be terminated if plasma HIV RNA determinations are >30,000 molecules/ml on two successive determinations at least 1 week apart.
2. At any time, Step II will be terminated if the circulating CD4+ T cell concentration falls below 250 cells/μL, or < 50% of the baseline determinations on two successive occasions performed at least 1 week apart.
3. Development of an intercurrent illness, which in the opinion of the investigator and/or PMD would contraindicate the discontinuation of HAART.
4. Non-adherence with study visits or medications during Step II, which in the opinion of the investigator would interfere with the subject's ability to complete the trial.

Following the termination of Step II, and the discontinuation of IL-2, and the reinstitution of HAART therapy, a subject will be followed every two weeks until viral load is <50 on two successive determinations.

If a subject and their respective physician decide not to restart HAART immediately after termination from Step II, their physician will assume sole responsibility for monitoring off HAART.

7.4 Step III – extension of Interruption of HAART

7.4.1 Inclusion

1. Completion of Step II, 12 weeks off HAART.
2. Plasma HIV RNA <30,000 at week 25.
3. CD4 + T cell concentrations >250 cell/µL or >50% of baseline.

7.4.2 Exclusion

1. Step III will be terminated if plasma HIV RNA determinations are >30,000 molecules/ml on two successive determinations at least 1 week apart.
2. Step III will be terminated if the circulating CD4+ T cell concentration falls below 250 cells/µL, or < 50% of the baseline determinations on two successive occasions performed at least 1 week apart.
3. Development of an intercurrent illness, which in the opinion of the investigator and/or PMD would contraindicate the discontinuation of HAART.
4. Non-adherence with study visits or medication during Step III, which in the opinion of the investigator would interfere with the subject's ability to complete the trial.

Following the termination of Step III, and the discontinuation of IL-2, and the reinstitution of HAART therapy, a subject will be followed every two weeks until viral load is <50 on two successive determinations.

If a subject and their respective physician decide not to restart HAART immediately after termination from Step III, their physician will assume sole responsibility for monitoring off HAART.

7.5 Study Enrollment Procedures

Screened subjects will read and sign part I of the consent form, which states that they agree to participate in the screening process to determine eligibility.

1. Once a candidate for study entry has been identified, details will be carefully discussed with the subject. The subject will be asked to read and sign the consent form (part II) that was approved by both the IRB and the DIAIDS, NIAID.

2. When a subject has been deemed ready for randomization, the Study Team will contact the Investigational Pharmacist, who will inform the Study Team whether the subject has been randomized to receive IL2 or not.
3. The randomization process utilized is that of a balanced blocked method and the software used to generate this randomization scheme is "Computer Programs for Epidemiologists", PEPI, version 3.00.

8 STUDY INTERVENTION

8.1 Drug Regimens, Administration, and Duration

All subjects will continue to receive potent antiretroviral therapy (two or more antiretroviral drugs in combination). In Step I, Subjects will be randomized to one of the following four treatment arms:

A. ALVAC placebo
B. ALVAC vCP1452
C. ALVAC-placebo + IL-2
D. ALVAC vCP1452 + IL-2

ALVAC vCP1452 or ALVAC placebo will be administered 1ml IM at weeks 0, 4, 8, and 12. The concentration may vary with each lot and will be specified on the label. The doses of ALVAC vCP1452 administered will contain ≥ $10^{6.5}$ $TCID_{50}$

IL2 will be administered as a subcutaneous dose of 1.2 MU/m2 daily beginning at week 0.

8.1.1 Timing of ALVAC vCP1452 or ALVAC placebo administration

All subjects (Arms A, B, C, and D) will receive ALVAC vCP1452 or ALVAC placebo at entry (week 0) and then at weeks 4, 8, and 12, plus or minus 14 days (2 weeks). Subjects must have received at least 4 vaccinations to be eligible for Step II.

8.1.2 Timing of IL2 administration

For subjects randomized to Arms C or D, IL-2 injections should be initiated within 24 hours of the injection of the vCP1452/ALVAC placebo.

8.1.3 IL-2 Preparation and Administration:

Materials:

IL-2 (18-million IU (MIU) vial)*
Preservative-free sterile water for injection USP 5% dextrose (D5W) injection.

*Nominal potency of 22 MIU/vial

Note: To facilitate reconstitution, dilution, and subsequent product withdrawal, the use of a locking luer adapter plug has been incorporated with successful results. The pharmacy should keep the pre-filled syringes refrigerated until the patient is ready to leave the clinic. Any unused portion of reconstituted vials and expired pre-filled syringes should be disposed of in accordance with institutional or pharmacy policy.

8.1.3.1 To prepare 3.6 million U/ml concentration so that each dose is 1.2 mU/M ^{2}:

Each vial should be reconstituted aseptically with 1.2 mL sterile water for injection, USP, preservative free. During reconstitution, the sterile water should be directed towards the side of the vial to avoid foaming (note: vials are packaged under a vacuum). Mixing should be accomplished with gentle swirling and/or slow inversion. DO NOT SHAKE. The resultant solution should be a clear, colorless to slightly yellow fluid. This yields an 18-million U/mL solution. To this vial, 4.8 ml D5W is added, yielding a solution of 3.6 mU/ml for injection. For an average adult, with 1.7 M^2 BSA, the volume to be administered x 1.2 mU/M^2 would equal 0.57 ml.

8.1.3.2 Il-2 Dosage:

The initial Il-2 dose will be at 0.9mU/m^2. If after receiving 14 days of IL-2 therapy at this dose there have been no systemic side effects (i.e. grade 0), subjects will be eligible for a 25% dose increase to 1.2 mU/m^2.

8.1.4 ALVAC vCP1452/ALVAC Placebo Preparation and Administration:

Prior to injection, each dose of vaccine or placebo is reconstituted by dissolution of the lyophilisate with the diluent supplied [i.e. 1 ml of sterile saline solution (NaCl 0.4%)]. The content of the vial containing the diluent will be aseptically withdrawn and injected slowly into the vial containing the lyophilized ALVAC. This vial will then be swirled gently until dissolution of the lyophilisate. Avoid inverting the vial. The reconstituted vCP1452 or ALVAC placebo must be kept refrigerated at 2° to 8°C (36° to 46°F) and used within two hours. Any unused portion of reconstituted vials and expired pre-filled syringes should be disposed of in accordance with institutional or pharmacy policy.

Each vaccine preparation is administered intramuscularly with a 22-gauge 1.5-inch needle. When ready for administration, the mixture should be swirled gently for 10 seconds. Administer the vaccine into the left deltoid muscle or upper thigh after preparation of the site with alcohol. Subjects must remain in the clinic for observations for 30 minutes after each immunization.

8.1.5 Drug Formulation:

8.1.5.1 Interleukin-2 (Proleukin, Aldesleukin, IL-2)

Aldesleukin (IL-2, interleukin-2, rhIL-2, rIL-2, Proleukin 7®) is supplied as a sterile, white to off-white, lyophilized cake in single-use vials. For the 18 million International Unit (MIU) (nominal potency of 22 MIU) vial, when reconstituted with 1.2 mL of sterile water for injection, USP (SWFI), each mL contains 18 MIU IL-2, 50 mg mannitol, and between 118-210 mcg sodium dodecyl sulfate (SDS), buffered with approximately 0.17 mg monobasic sodium phosphate, buffered with approximately 170 mcg sodium phosphate dibasic monohydrate and 890 mcg sodium phosphate to a pH of 7.5 (range 7.2 to 7.8). Unopened vials must be stored in a refrigerator at 2°C to 8°C (36°F to 46°F). Do not freeze.

8.1.5.2 ALVACvCP1452[ALVAC(2)120(B,MN)GNP(vCP1452),ALVAC(2)HIV (vCP1452)]

ALVAC vCP1452 is supplied as a sterile, lyophilized product in single-dose vials. The concentration may vary with each lot and will be specified on the label. The diluent supplied for reconstitution of vCP1452 consists of a sterile solution of NaCl 0.4%, which is adjusted to a pH of 4.5 to 7 and conforms to established requirements for sterility, safety, and pyrogen testing. Store the lyophilized vaccine at 2°C to 8°C (36°F to 46°F) and use within two hours after reconstitution. Do not freeze.

8.1.5.3 ALVAC-placebo

ALVAC placebo is supplied as a sterile, lyophilized product in single-dose vials containing a mixture of 10 mM Tris-HCl buffer pH 9.0 (1/4 volume), virus Stabilizer (1/4 volume) and freeze drying medium (1/2 volume). The diluent supplied for reconstitution of ALVAC- Placebo consists of a sterile solution of NaCl 0.4%, which is adjusted to a pH of 4.5 to 7 and conforms to established requirements for sterility, safety, and pyrogen testing. Store the lyophilized product at 2°C to 8°C (36°F to 46°F) and use within two hours after reconstitution. Do not freeze.

8.1.6 Drug Supply, Distribution, and Pharmacy

Chiron Corporation will provide IL-2. Aventis Pasteur will provide vCP1452 and ALVAC-placebo. Guidance for handling study agents is available in the *Pharmacy Guidelines and Instructions for the DAIDS Clinical Trials,* in the section Investigational Agent Control.

8.1.7 Investigational agent accountability

The protocol Investigational Pharmacist is required to maintain complete records of all study medication received and subsequently dispensed. All unused study medication must be returned after the study is completed or terminated. The types

of procedures to be followed are provided by a DAIDS manual, Pharmacy *Guidelines and Instructions for,* in the section Investigational Control.

8.2 Concomitant Medications:

8.2.1 Permitted Medications:

1. Topical corticosteroids: topical corticosteroid use is acceptable provided it is applied to a site separate from IL-2 and vaccine injection sites. .
2. Maintenance therapy for opportunistic infections that develop on study treatment is permitted according to standard medical care, except for foscarnet during IL-2 administration, and rifabutin and rifampin at any time.
3. Maintenance therapy for recurrent genital herpes with ≤ 1000 mg/day of acyclovir is permitted.
4. Erythropoietin and filgrastim (G-CSF) are permitted when clinically indicated.
5. All antibiotics for bacterial infections as clinically indicated.
6. Medications for symptomatic treatment such as antipyretics and analgesics. Ibuprofen and acetaminophen are the preferred agents.
7. Hypothyroidism: Concomitant use of thyroid hormone replacement is permitted to manage hypothyroidism.

All concomitant drugs should be recorded in the database.

8.2.1.1 Antiretroviral Treatment Modifications for Changes in Viral Load

During Step I, adjustments in the protease inhibitor or nucleoside analogues will be permitted if for reasons other than virologic failure.

8.2.2 Prohibited Medications:

Concomitant medications prohibited during the course of this study include:
1. interferons
2. interleukins (other than the study agent IL-2)
3. HIV vaccines (other than the study agent ALVAC vCP1452)
4. sargramostim (GM-CSF)
5. dinitrochlorobenzene (DNCB)
6. thymosin alpha 1 (thymosin alpha)
7. thymopentin
8. inosiplex (Isoprinosine)
9. polyribonucleoside (Ampligen)
10. ditiocarb sodium (Imuthiol)
11. investigational antiretroviral agents
12. thalidomide
13. St. John's wort
14. systemic or local cytotoxic chemotherapy for malignancy
15. systemic corticosteroids (see 6.411 for further details)

8.2.3 Adherence Assessment

Adherence to antiviral medications in Step I will be assessed by monitoring the plasma HIV concentration. Adherence to the ALVAC vCP1452/placebo will be assessed by direct observation of the Study Team in the GCRC. Adherence to the self-injection of IL2 will be assessed by observation of the inflammatory reactions at the sites of injection, and by collection of used syringes.

9 CLINICAL & LABORATORY EVALUATIONS

9.1 Schedule of Events: (Please refer to Evaluations Table, Appendix)

9.1.1 Screening Visits (SV 1 & 2):

Screening visits will take place at the study sites within 14-30 days prior to week –2. During these visits, the study will be explained and discussed in detail and a copy of the Study Informed Consent Form will be provided to the subject to take home.. Written informed consent will be obtained to perform screening laboratory evaluations (subjects will sign part I of informed consent form). A complete medical history and physical examination will be performed if the subject is interested in the study at SV #2.

Definitions of evaluations given below:

Screen Visit # 1:

1. Discussion of informed consent
2. NYPH Labs, including:

    a. HIV Antibody test by Elisa/ Western Blot Analysis (1 red tube-5 mL)
    b. Ultrasensitive viral load (1 white tube-5 mL)
    c. CBC, platelets, differential (1 small lav tube-3 mL)
    d. Lymphocyte subsets (2 small lav tubes-6 mL)
    e. CMP/Chol (Comprehensive Metabolic Profile/Cholesterol) (1 yell tiube-5 mL)
    f. TSH (CMP tube)
    g. Hepatitis Profile (A, B, C). (CMP tube)
    h. Urine pregnancy test (women only)
    i. IL-2 antibody test (only for subjects with prior IL-2 therapy) (1 yell-5 mL)

Screen Visit # 2:

Continued discussion of study

Labs, including:

a) Ultrasensitive viral load (1 white-5 mL)
b) Lymphocyte subsets (2 small lav-6 mL)
c) CBC, platelets, differential (1 small lav-3 mL)

Repeat of any abnormal labs from first visit

Complete History & Physical examination, including body surface area (BSA)

After evaluation of results, the subject will be notified of their eligibility by phone and if the subject is interested they will be invited to return to the study site for a randomization visit.

9.1.2 Randomization & Baseline Visits (weeks -2, -1, 0)

Randomization will occur at week –2, and at weeks –2, -1 and 0, baseline laboratory values will be determined.

At visit 3 (week –2), the subject will meet the entire study team, including the Protocol Chair, and the study will be discussed, particularly the risk/benefit ratio, the concept of voluntary participation, and protocol compliance. If the subject wishes to participate, Part II of Study Informed Consent Form will be signed and witnessed. A copy of the signed consent form will be given to the subject to keep. The subject will then be enrolled into the study, and randomized by the Clinical Trial Coordinator, who will contact the Protocol Pharmacist by phone.

Three baseline evaluations will be performed 1 week apart prior to beginning study treatments.

Laboratory and physical examination will be performed according to the timetable. Where an X is indicated in the table, the appropriate physical and laboratory evaluations will be performed.

1. Randomization: subjects will be randomly assigned into one of 4 groups below

Group	IL-2	Vaccine
A	No	Placebo
B	No	Vaccine
C	Yes	Placebo
D	Yes	Vaccine

Table III: Randomization groups

2. Interim history
3. The following labs will be drawn at week –2:
    a. Ultra- sensitive viral load
    b. Lymphocyte subsets
    c. CBC, platelets, differential
    d. Three large heparinized tube (7ml green top) for CPLp assay
    e.
    f. Two large heparinized tubes (7ml green top) for immunology studies (@ week –1 only)

g. ELISPOTs-3 lg lav tubes (30 mL)
h. Urine pregnancy test (women only)

3. The following labs will be drawn at week –1:
   a. Ultrasensitive viral load (1 white-5 mL)
   b. CBC, Diff, platelets (1 small lav-3 mL)
   c. Lymphocyte Subsets (2 small lav-6 mL)
   d. Plasma-(7ml green top) spun and frozen at -80º
   e. Pathology Tests-(3 green-21 mL).
4. The following labs will be drawn at **visit 5, week 0,** prior to vaccination or IL-2 injection:
   a. Ultrasensitive viral load (1 white-5 mL)
   b. Lymphocyte subsets (2 small lav-6 mL)
   c. CBC, platelets, differential (1 small lav-3 mL)
   d. Thyroid function (TSH) (Only for IL-2 recipients) (CMP Tube)
   e. CMP/Chol

   f. Urine pregnancy test (women only)
   g. CPLp Assay-Three large heparinized (21ml green top)

Immunology Assays-4 large heparinized tubes (28ml green top)

5. Vaccine/Placebo vaccine will be administered at **visit 5, week 0**.
6. If in groups C or D, IL-2 injection will be given at visit 5, week 0, and subjects will be taught to give themselves injections.
7. Subjects in groups C and D will return every two weeks to pick up a supply of IL-2.

9.1.3 Step I, Vaccination-weeks 4 # 8, and 12:

1. The following labs will be drawn prior to each vaccination/placebo:
   a. Ultrasensitive viral load (1 white-5 mL)
   b. CBC, Diff, plate (1 small lav-3 mL)
   c. Lymphocyte subsets (2 small lav-6 mL)
   d. TSH (Only for IL-2 recipients) (CMP tube)
   e. CMP/Chol (1 yell-5 mL)

   f. Urine pregnancy test (women only)
   g. CPLp AssayThree heparinized tube (7 ml green top)
   h. Plasma-One large heparinized tubes (7ml green top) spun and frozen at -80° for plasma banking (@ week 12 only)
   i. Immunology Assays-4 large heparinized tubes (7ml green top) for immunology studies (@ weeks 4 & 12 only)

2. Vaccine/Placebo will be administered
3. Problem-oriented history and physical
4. Phone visit will be scheduled

9.1.4 Post vaccination weeks-#s 5, 9 and 13

1. The following labs will be drawn 1 week after each vaccination:
    a. Ultrasensitive viral load (1 white-5 mL)
    b. CBC, Diff, Plates-(1 small lav-3 mL)
    c. Lymph Subsets-(2 small lav-6 mL)
    d. CPLp (3 green-21 mL)
    e. ELISPOT-(3 lg lav-30 mL)

9.1.5 Week 12.5 Phone visit:

1. Labs will be reviewed with patient
2. Development of new exclusionary criteria will be reviewed.
3. Day of start of the DTI will be scheduled. If the subject is on Sustiva, that medication will be discontinued 72 hours prior to the discontinuation of other antiviral medications and if subject is on Viramune, that medication will be discontinued 48 hours prior to the discontinuation of other antiviral medications.
4. Documentation of phone visits and date of HAART discontinuation to be recorded in patient's record.

9.1.6 Week 13: Begin Step II:

1. Targeted history and physical
2. Laboratory evaluation as follows:
    a. Ultra-sensitive viral load
    b. CBC, platelets differential
    c. Lymphocyte subsets
    d. Three heparinized tube (7 ml green top) for CPLp assay

9.1.7 Weeks 14-16:

1. Targeted history and physical
2. Laboratory evaluation as follows:
    a. Ultra-sensitive viral load (1 white-5 mL)
    b. CBC, platelets, differential (1 small lav 3 mL)
    c. Lymphocyte subsets (2 small lav-6 mL)

9.1.8 Week 17):

1. The following labs will be drawn:
    a. Ultra-sensitive viral load (1 white-5 mL)
    b. CBC, platelets, differential (1 small lav-3mL)
    c. Lymphocyte subsets (2 small lav-6 mL)
    d. CMP/Chol (1 well-5mL)
    e. TSH (CMP tube)

   f. Urine pregnancy test (women only)
   g. Immunology-(4 green-28 mL)

2. Problem-oriented history and physical

9.1.9 Weeks 18-20), Weeks 22-24) and bi-weekly during Step III(wks 25-36)

1.
2. Laboratory evaluation as follows:
   a. Ultra-sensitive viral load (1 white-5 mL)
   b. CBC, platelets, differential (1 small lav-3 mL)
   c. Lymphocyte subsets (2 small lav-6 mL)
   d. Two white top tubes for viral resistance (@ week 20 only)
   e. One large heparinized tubes (7ml green top) to be spun and frozen at -80º for plasma banking (@ week 24 only)
   f. Immunology Assays-4 large green top; 28 mL) (@ week 24 only)

9.1.10 Weeks 21 & 25

1. The following labs will be drawn:
   a. Ultra-sensitive viral load (1 white-5 mL)
   b. CBC, Diff, Plate-(1 small lav-3 mL)
   c. Lymphocyte subsets (2 small lav-6 mL)
   d. CMP/Chol (1 yell-5 mL)
   e. TSH (CMP tube)

Urine pregnancy test (women only)
   f. CPLp assay-(3 lge green-21 mL)
   g. Immunology studies (4 green-28 mL)(@ week 21 only)
   tubes (7ml
2. Problem-oriented history and physical

Note: 4 large heparinized green top) for immunology studies will be drawn 2 weeks after the first detectable viral load in Step II – week number will vary based on time of detectable viremia.

9.2 Weeks 26 – 36 Step III

1. The following labs will be drawn:
   a. Ultrasensitive viral load (1 white-5 mL)
   b. Lymphocyte subsets (2 small lav-6 mL)
   c. CBC, platelets, differential (1 small lav-3 mL)

   And in addition at weeks 29, 33, 37

   d. CMP/Chol-1 well-5 mL)
   e. TSH (CMP tube)
   f. CPLp, Viral Resistance & plasma (week 37 only)-(5 green-35 mL)

   g. Urine pregnancy test (women only)
2. Problem- oriented history and physical-(Week 37 only)

9.3 Definitions for Schedule of Events:

Pretreatment Evaluations

Evaluations that occur prior to the subject taking any study medications, treatments or interventions are defined as "pre-treatment evaluations". All pre-treatment evaluations must occur no more than 30 days prior to study entry. The screening evaluations are defined as pre-treatment evaluations.

On Study Evaluations:

Evaluations should occur after randomization. Study visits must be scheduled on the weeks (± 7 days) indicated.

Evaluations for randomized subjects who never begin study treatment:

Subjects who are randomized but who never begin treatment will not undergo study treatment evaluations.

Treatment Discontinuation Evaluations:

Subjects who permanently discontinue study treatment for any reason prior to completion of week 36 will continue to be followed on study, off treatment with all evaluations.

Post-Treatment Off Study Evaluations (Week 36):

Subjects who prematurely discontinue treatment will return to the clinic at the time of permanent treatment discontinuation and will continue to be followed on study, off treatment until week 36, at which time there will be a final on study, off treatment evaluation.

Informed Consent:

A signed and dated, IRB-approved informed consent is required prior to study entry.

Medical/Medication History:

The study team will obtain a medical history and medication history at the time of the screening visit. The medical and medication history will include a complete HIV-related diagnosis; psychiatric history, particularly any history of depression requiring therapy, current or recent use of illicit drugs, and history of alcohol abuse

or dependence; any prescription medications taken within the last 35 days; current contraception method if participating in sexual activity that could lead to pregnancy; and any allergies to any medications.

Study Treatment Modifications:

All modifications to study drugs including initial doses, protocol-mandated interruptions, modifications, and permanent discontinuation of treatment will be recorded in the database by the Study Team at each visit. Any changes in study medications (e.g. dose changes, dose interruptions, discontinuation of study drugs) will be authorized by the study team, and noted in the database by the Clinical Trials Coordinator.

Complete Physical Exam:

A complete physical exam is required at screening and when clinically indicated. The physical exam should include: any signs and symptoms experienced within 30 days prior to study entry, weight, height, temperature, blood pressure, Karnofsky status, pulse rate, and respiration.

Targeted Physical Exam:

A targeted physical examination, to be driven by any signs or symptoms previously identified that the subject has experienced since the last visit, and defined as signs, symptoms, and diagnoses.

Signs & Symptoms:

All signs, symptoms and toxicities must be recorded in the database.

Diagnoses:

All confirmed and probable diagnoses made since the last visit will be recorded in the source documentation, including current status at the time of study visit.

NYPH Laboratory Evaluations:

All laboratory values must be recorded in the database.

Ultrasensitive Viral Load:

The determination of plasma HIV RNA concentration will be performed using the Roche PCR-based assay, which has an LLD of 50 HIV RNA mol/mL.

This assay will be performed at the NYPH labs.

Hematology:

Hemoglobin, absolute neutrophil count (ANC), platelets, CBC with differential.
These evaluations will be performed at the NYPH labs.

Record all Hgb, ANC, and platelets in database and enter the values in real time within 48 hours.

Lymphocyte Counts:

Specific lymphocyte counts of T, B and NK cells will be performed at the NYPH labs. They use the methods that have been certified by the Pathology Department @ NYPH, which entail flow cytometry using Becton Dickinson (BD) monoclonal antibodies and B D flow cytometers.

Comprehensive Metabolic Profile(CMP):

Glucose, electrolytes [e.g., sodium, potassium, chloride, bicarbonate], serum creatinine, BUN. These evaluations will be performed at the NYPH labs.

Record all values in the database, and enter values real time into the database within 48 hours.

Liver Function Tests (LFT):

AST (SGOT), ALT (SGPT), alkaline phosphatase, total and direct bilirubin.
These evaluations will be performed at the NYPH labs.

Record all values in the database, and enter values real time into the database within 48 hours.

Hepatitis Profile:

Sera will tested for antibodies reactive with HAV, HBV, and HCV. These evaluations will be performed at the NYPH labs.

Record all values in the database, and enter values in real time within the database within 48 hours.

Thyroid Stimulating Hormone:

TSH levels will be performed at 4-week intervals. The NYPH labs will perform these evaluations.

Record all values in the database, and enter values real time into the database within 48 hours.

Pregnancy Test:

For women with reproductive potential: Serum or urine β-HCG (urine test must have a sensitivity of 50 mIU/mL).

This evaluation will be performed at the New York Presbyterian Hospital (NYPH) Laboratories.

Research Lab Studies:

Cytokine Producing Lymphocyte precursors (CPLp):

This assay uses flow cytometry to identify the lymphocyte subsets (CD3, CD4, CD8 and CD56/CD16) and to identify the expression of intracellular cytokines (IL-2, IFN-α and TNF-α) in response to activation by HIV peptides. If all HIV peptides are not available by the initiation of the study, PBMCs will be obtained at the times indicated and frozen at a fixed rate of -1° C/min., then kept in liquid nitrogen until peptides become available.

These evaluations will be performed at the Weill Cornell Immunology Division Research Labs.

Viral Resistance Testing:

Plasma frozen at -80° C will be tested for phenotype and genotypes characteristic for resistance to approved antiviral agents. The NYPH labs will perform these assays.

Immunology Studies:

Heparinized blood will be obtained at the defined time points and evaluated for:

1. flow cytometery for surface and intracellular expression of proliferation (Ki-67, Bcl-20, apoptosis (annexin-V, activated caspase –3) and other specific markers (CD25, CD45RA, CCR7, CD57) and NK cells will be evaluated for expression of perforin, granzymes and cytotoxic activity.
2. Isolation of HIV –specific T cells using magnetic beads based on interferon-gamma expression after short-term stimulation. Isolated T cells will be cultured and evaluated for proliferative capacity and interleukin 2 production.

Pathology Assays:

BlyS, APRIL and antibody production will be assayed on heparinized blood.

ELISPOT Assays:

EDTA-anticoagulated blood will be obtained for assays of interferon-γ-producing cell frequencies after activation o/n with HIV peptides to achieve an estimate of the frequency of HIV-reactive PBMCs, and to compare with the data obtained via CPLp assays.

10 TOXICITY MANAGEMENT

10.1 Description and management of adverse reactions

10.1.1 Study Treatment Discontinuation for Toxicity

10.1.1.1 IL2

Adverse reactions expected with low-dose IL-2:
Local injection site reactions – erythema and/or induration at injection site, subcutaneous nodules, pruritus
General/Constitutional – fever, chills, arthralgia, myalgia, fatigue, flu-like syndrome, weakness, worsening of pre-existent inflammatory or autoimmune disease.
Cutaneous – rash, pruritus,
Respiratory – nasal congestion, allergic rhinoconjunctivitis, asthma exacerbation,
Gastrointestinal – nausea, vomiting, diarrhea, dyspepsia
Endocrine – abnormal thyroid function
Neurologic – dizziness, headache
Hematologic – anemia, thrombocytopenia, neutropenia, eosinophilia

A case of myocardial infarction, pneumonia and optic neuritis was reported in the previous study of low-dose IL-2. IL-2 could not be ruled out as causative.

Immediate hypersensitivity reactions including anaphylaxis were rarely observed after the administration of IL-2.

Other adverse events have been observed in studies using higher doses of IL-2 administered as intermittent subcutaneous injections or IV infusions. They are not expected with the low-dose IL-2. Refer to the Investigator's Brochure for the description of such reactions.

IL-2 should be permanently discontinued for any study drug-related Grade 4 toxicity except hematological (which can be corrected with EPO or G-CSF or blood transfusions). If at any time during the study, ALT is $\geq$ 20 times the upper limit of normal, both IL-2 and immunization intervention should be permanently discontinued in that subject.

Electrolyte abnormalities that can be readily corrected will not require permanent drug discontinuation.

10.1.1.2 IL2 Dosage modifications:

Subjects receiving IL-2 may have their IL-2 interrupted for toxicity:

1. Electrolyte abnormalities Grade 1 or higher that cannot be rapidly corrected.
2. Grade 1 or higher respiratory toxicity.
3. Grade 3 local reactions or any local reaction involving ulceration.
4. Fever > 39 ^{o}C, or intolerable flu-like symptoms or rigors.
5. Other Grade 3 or greater toxicity either related or unrelated to IL-2.
6. Fever suspected to be related to an opportunistic infection.
7. Grade 2 fatigue.
8. Grade 2 hematological toxicity that can be corrected with EPO, G-CSF, or blood transfusions.

Subjects who have their IL-2 interrupted can have it resumed at the same dose or at one dose level reduction within 24-48 hours of stopping drug. The dose of IL-2 for that subject may later be increased to the initial dose at the discretion of the subject and the local investigator.

10.1.1.3 Reactions thought definitely, possibly, or probably related to study vaccines

10.1.1.3.1 <u>Local reactions to vaccine:</u>

- Local reactions will be graded using the NIAID Division of AIDS (DAIDS) scales (Table 3).
- Discomfort will be graded according to the DAIDS Pain and/or Tenderness Grading Scale.

Cutaneous reaction will be graded according the DAIDS Serious Adverse Event Reporting System (cutaneous).

It is anticipated that many recipients of vCP1452 or ALVAC placebo may experience transient tenderness, redness, or swelling at the local site of injection. Participants should be told prior to administration that these anticipated reactions might occur and the participants must remain at the clinic for observation for at least 30 minutes after each immunization.

10.1.1.3.1.1 <u>Local reactions Grade 1 or 2 on the DAIDS Scales</u>

Local reactions of mild (Grade 1) or moderate (Grade 2) severity will usually resolve spontaneously. If needed, they may be managed with local application of

cold packs, oral acetaminophen, oral non-steroidal anti-inflammatory agents, or a combination of these measures as appropriate.

10.1.1.3.1.2 Local reactions Grade 3 or 4 on the DAIDS Scales

All Grade 3 or 4 local toxicities should be considered for modification or discontinuation of immunizations.

For grade 4 local reactions, definitive medical and/or surgical intervention should be undertaken as appropriate. Further vaccines should not be given prior to consultation with the protocol chair. With approval, the vaccines might subsequently be administered at less frequent intervals.

10.1.1.3.2 Systemic reactions

Systemic reactions will be graded according to the Common Toxicity Criteria Table from the Cancer Therapy Evaluation Program.
The Principle Investigator should be contacted within 48 hours for any non-local Grade 3 or 4 reactions (for example, elevated temperatures following immunizations) thought definitely, possibly, or probably related to vaccination with vCP1452 or their placebos.
Further vaccines should not be given prior to consultation with the Principle Investigator. In consultation with the Principle Investigator, the NIAID, the Corporate Sponsors, and the FDA, the decision will be made whether the vaccine/placebo should be administered less frequently or discontinued.

Grade	DAIDS<br>Pain and/or tenderness<br>Grading system	DAIDS<br>Serious Adverse Event Reporting System<br>*Cutaneous*
0 = None	No pain or tenderness	N/A
1 = Mild	Minimal pain or tenderness, no limitation of arm use	Erythema or induration<br>Less than 15 cm x 15 cm (225 cm sq)
2 = Moderate	Notable pain or tenderness, some limitation of use of arm	Erythema, induration or edema ≥ 15 x 15 cm (225 cm sq)
3= Severe	Extreme pain or tenderness, complete limitation of use of arm	Ulceration or superinfection or phlebitis
4= Potentially Life-threatening	N/A	Necrosis of the skin

10.2 Reinstitution of HAART

10.2.1 Low CD4+ T cell Concentrations

10.2.1.1 Step I

Subjects on Step I who have a CD4 count < 250 cells/mm^3 or less than 50% of baseline on two consecutive occasions will be discontinued from receiving study drugs and will be followed on-study, off-treatment.

10.2.1.2 Step II

Subjects on Step II who have a CD4 count < 250 cells/mm^3 or <50% of baseline on 2 successive occasions will be terminated from Step II and recommended to immediately resume HAART. After resuming HAART, these subjects will be followed every two weeks until CD4 T cell counts are above 250/mm^3 or > 50% of baseline.

10.3 Criteria for Study Discontinuation:

Subjects will be withdrawn from the study for the following reasons

1. Noncompliance: The subject takes no study medications (IL-2 and/or vaccine and/or placebo) or discontinues these medicines following randomization to Step I.
2. The subject is non-adherent with clinical visits
3. Treatment-related toxicity that requires permanent study treatment discontinuation.
4. Required use of any prohibited medications.
5. The subject voluntarily withdraws from the study for any reason.
6. Pregnancy and breast-feeding are reasons for immediate permanent discontinuation of study drugs, and appropriate referrals should be arranged. The subject will be followed for safety for at least 1 month. Fetal outcome will be recorded in the clinic chart (if it is possible to obtain this information).

11 STATISTICAL CONSIDERATIONS

11.1 General design Issues

This is a phase II, randomized, partially double-blind, single center, 2x2 factorial experiment with four arms: one in which no immunotherapeutic approach is administered, one in which subjects receive a canarypox vector HIV vaccine, one in which subjects receive daily low dose subcutaneous IL2, and one in which subjects receive both the canarypox HIV vaccine and daily low dose subcutaneous IL2. IL2 will be given open label, while the canarypox HIV vaccine will be placebo-controlled and double- blinded. All subjects will receive HAART during the immunotherapeutic interval.

The treatment comparisons of most interest are:

- HIV vaccine vs. control
- IL2 treatment vs. control

- HIV vaccine + IL2 treatment vs. control
- HIV vaccine + IL2 treatment vs. either HIV vaccine or IL2 treatment alone.

11.2 Primary Endpoints:

Antiretroviral therapy will be interrupted for a minimum of 12 weeks, and the primary endpoints for evaluating immune control of viral replication will be 1) the proportion of subjects that remain aviremic during Step II, 2) the mean of the $\log_{10}$ (HIV RNA molecules/ml) obtained from weekly blood samples during on-study weeks 21-25, which correspond to weeks 8-12 following the cessation of HAART, and 3) the proportion of subjects among each group who are eligible to progress to Step III. Means will be compared using analysis of variance (ANOVA) for a 2x2 factorial design. Initially, the vaccine x IL2 interaction term will be evaluated in order to determine whether the difference in viral load response between vaccine and control varies according to whether or not IL2 was administered. If the interaction is significant, then specific "within level" contrasts will be carried out. A Fisher’s exact test will be used to compare proportions between the 4 experimental groups.

11.3 Sample Size & Power for the Primary Endpoints:

Assuming that 90% of the subjects randomized to the control arm (i.e. no immunotherapeutic intervention) relapse by week 12, if any of the experimental immunotherapies were to reduce the incidence by half, i.e. to 45%, then 21 subjects per group would be necessary to detect this reduction with 90% power using a 2-tailed chi square test, @ 5% significance level.

We anticipate enrolling 23 patients per arm with an anticipated dropout rate of about 25%, resulting in 17 patients per arm. Based on our previous open label study using daily low dose subcutaneous IL2 administration both before and after the cessation of HAART, the mean plateau value of the “trough” plasma HIV RNA molecule concentration was 4.2 ± 0.5 (SD) $\log_{10}$. Using a Bonferroni-adjusted t-test approach to the power calculation for comparing each of the three active treatment arms to control (alpha=0.05/3 =0.017), this yields 80% power for detecting a difference of 0.60 log mol/ml or 90% power for detecting a difference of 0.65 log units.

11.4 Secondary Endpoints:

1. The duration that subjects remain off of HAART following the cessation of antiretroviral therapy. The Kaplan-Meier product limit method and the log rank test will be used to estimate and compare time until relapse among the four treatment arms. The relapse rate specifically for 12 weeks will be computed from the KM curve. The more general question of comparing overall times until relapse will be determined from the KM curve based on the total follow-

up of all patients in the study. Any patient dropping out or who fails to relapse by the time of statistical analysis will be considered censored.

2. The concentrations of circulating lymphocyte subsets (i.e. CD4+ T cells, CD8+ T cells, NK cells), their state of activation, and the frequency of HIV-specific T cells as assessed by flow cytometry measuring the expression of activation markers after a short-term activation *in vitro* within the first 12 weeks following cessation of HAART. These parameters will also be studied using 2x2 ANOVA, with data transformations, as indicated.

11.5 Sample Size & Power for the Secondary Endpoints:

Analysis of the immunologic secondary endpoints will be exploratory since the sample size of this study is not large enough to provide both sufficient protection against a type I error and adequate power to test more than the hypotheses about the primary endpoint. The differences between the immunologic assays will be explored by using 95% confidence intervals to estimate the difference between each of the treatment arms and the control arm.

11.6 Interim Analysis and Early Stopping

There will be one interim analysis for efficacy, which may result in early termination of the trial. The interim analysis will take place after half of the projected sample size has been followed for the required 25 weeks. Thus, the interim analysis can be performed when 34 subjects have completed step II (calculated based upon a drop-out rate of 25%, yielding 92 enrolled subjects X 75%/2 = 34. At that time, the 2x2 factorial ANOVA will be carried out. Using a Lan-DeMets version of an O'Brien-Fleming group sequential stopping rule, the trial will be stopped if at least one of the three tests (i.e., main effect for vaccine, main effect for IL-2, or interaction) is statistically significant at the alpha=0.00305 significance level. If none of the tests are significant, then the trial will continue until the planned termination with 92 subjects enrolled and 68 subjects having completed Step II The final ANOVA will be carried out at the alpha=0.04695 significance level. This rule will preserve an overall 5% significance level and will also preserve the projected statistical power.

It should be noted that the application of an early stopping rule to other than a simple two-sample problem can be problematic. In this trial, there are three statistical tests being carried out in the 2x2 ANOVA. The approach here was to handle the interim analysis of the three tests in much the same way as the three tests are handled in a standard ANOVA problem – that is, just apply the appropriate P-value criterion to each of the three tests. In this case, the appropriate P-value is derived from the Lan-DeMets alpha spending function.

12 ADVERSE EVENT REPORTING

The reporting of Serious Adverse Events (SAEs) will be done so in accordance with 21 CFR 312.32. Adverse experiences occurring during treatment and the immediate 16-week period after the last dose of study treatment that meet SAE reporting requirements must be reported to the NAID, IRB, the FDA, and the study corporate sponsors.

A serious adverse drug experience: Any adverse drug experience occurring at any dose that results in any of the following outcomes:

- Death
- A life-threatening adverse drug experience
- Inpatient hospitalization or prolongation of existing hospitalization
- A persistent or significant disability/incapacity
- A congenital anomaly/birth defect
- Important medical events that may not result in death, be life-threatening, or require hospitalization may be considered a serious adverse drug experience when, based upon appropriate medical judgment, they may jeopardize the patient or subject and may require medical or surgical intervention to prevent one of the outcomes listed in this definition.

Unexpected adverse drug experience: Any adverse drug experience, the specificity or severity of which is not consistent with the current investigator brochure; or, not consistent with the risk information described in the general investigational plan or elsewhere in the current application.

The sponsor and the Independent Safety Monitor who is a member of the SMC can make the assessment of relatedness: This is not solely the responsibility of the investigator. If at any time an assessment of relatedness is made on an adverse event that is both serious and unexpected, it must be reported in an expedited fashion (i.e., "All cases judged by either the reporting health care professional or the sponsor as having a reasonable suspected causal relationship to the medicinal product qualify as adverse drug reactions (ADRs) in ICH ***Clinical Safety Data Management: Definitions and Standards for Expedited Reporting,*** **"** October 27, 1994, adopted by FDA in March, 1995").

The sponsor shall notify FDA by telephone or by facsimile transmission of any unexpected fatal or life-threatening experience associated with the use of the drug as soon as possible but in no event later than 7 calendar days after the sponsor's initial receipt of the information.

The study will be stopped based on the development of >20% of serious or unexpected adverse events.

13 DATA COLLECTION/MANAGEMENT

13.1 Overview

The Informatics Core of the General Clinical Research Center at the Weill Medical College of Cornell University will have primary responsibility for the development of the data management system and procedures for this trial. Support will be provided in database programming, database query, data quality assurance, report production, and auditing.

The proposed database will be centrally located on a Windows NT server at the Weill Cornell Informatics Core, with a web-based interface allowing data entry and data access from offices within Weill Cornell as well as from outside locations. Confidentiality will be maintained via secure socket layer (SSL) connections, password protection, and the absence of individually identifiable information (patient names, history numbers, social security numbers, etc.) in the database. Keys linking ID's to patient identifying information will be kept in a separate, secure location in the PI's office area.

13.2 Database Design and Programming

Data will be stored in a Microsoft Access database. The web-based "front-end", written in ColdFusion, JavaScript and HTML, will provide mechanisms for entering, viewing, printing, and exporting data. The data-entry screens will be set up to allow for data-entry by the study team during the clinic visit, and the system will include features to ensure that all necessary tests and examinations are performed at each visit (i.e., the physician will be able to pull up a list of requirements for the current visit number, and will be alerted if a required test or exam has not been completed).

Each element in the database will be defined with respect to data type (e.g. numeric, alphanumeric, choice field), data length and format. Data fields, which represent a choice from a predetermined list, will be coded as "choice fields."

The system will include features for assuring high-quality data, namely: range checks for numeric fields, validity and consistency checks (e.g. date fields), and other internal audits.

13.3 Data Entry Procedures

Clinical data (physical examination, vital signs, etc.) will be entered manually into a networked PC at each clinic visit. Laboratory data will be imported from the hospital lab database and the investigator's lab database. Built-in range-checking will assist in flagging unlikely clinical data at the time of entry. An audit trail system will record each record addition, deletion or modification together with the identity of the individual making the change.

13.4 Data Quality Assurance: Queries and Audits

Data quality assurance is accomplished in two formal ways: Data validation during data-entry (described above), and database query generation.Database query generation involves querying the database to identify data inconsistencies (e.g., dates in wrong order), potentially invalid values, etc. A specially formatted report is generated and reviewed by the clinical data collection personnel for resolution. This procedure is repeated on a regular basis (e.g., monthly) throughout the course of the study.

13.5 Confidentiality

Throughout the study, patient information will be kept confidential. Only patient ID numbers and initials will be used to identify subjects in the database. Although there will be a record to their true identities, only the investigators and staff on the project will have access to this record, which will be physically separate from the web-based database and maintained in a locked location. All printed reports will be kept in a locked file cabinet and strict password control will be used to limit and restrict database access.

Data Security

Currently, the Weill-Cornell Informatics Core has in place a well-secured computer network environment. Informatics Core staff and investigators have restricted access to directories and files in the server, according to their project responsibilities. This will be achieved primarily through the Windows NT Server security and the ColdFusion front-end, which have the capability to define different levels of security, as specified by the network administrator. Login/password security is used to control access to the specific web pages. The user's login and password must be valid and must be granted access to a page by the network administrator, or else access is denied.

13.6 Data Backup Procedures

All data are backed up each business day to tape. One tape per week is removed from the Informatics Core and stored at a remote location. The Informatics Core has considerable experience in setting up and implementing backup procedures and will have this responsibility in this project.

13.7 Hardware and Software

The Informatics Core's Windows NT server is a Hewlett Packard Netserver with RAID 5 hot-swappable hard drives and a matching backup domain controller (BDC). Both primary and backup servers are configured with one-button disaster recovery.

The General Clinical Research Center (GCRC) will provide examination rooms for clinic visits. Each examining room will have a networked PC capable of accessing this project's database via its web browser. The Informatics Core maintains a high-speed networked laser printer for use in printing clinic visit summaries and other reports.

14 SAFETY MONITORING

A Safety Monitoring Committee, formed according to the NIAID guidelines, will review the progress of each subject enrolled on the protocol. The responsibilities of this committee will be:

1. To be familiar with the research protocol and plans for safety monitoring
2. To review interim data to determine whether the trial should continue as designed, should be changed, or should be terminated.

An Independent Safety Monitor (ISM) will be a member of the Safety Monitoring Committee. The ISM will be provided with monthly reports on all adverse events. The ISM will be contacted immediately or at least within 5 working days of the occurrence of the event or within 5 days of the investigators knowledge of the occurrence of the event.
The SMC will meet regularly, to conduct reviews of the study progress and of accumulated safety data. The frequency of these meetings will be determined by the SMC. A suggested interval between meetings is 3 months or at the discretion of the ISM.

Our proposed plan for safety monitoring is that in the first 12 weeks of the study, during Step I, particular attention should be focused on the safety of the immune-based therapies. During Step II, when the antiviral drugs are withdrawn, particular attention should focus on the concentration of circulating CD4+ T cells. After the reinitiation of HAART, the SMC should focus on the restitution of each subject to undetectable plasma HIV RNA concentration. The ISM and SMC will meet at or very close to trial initiation in order to review the monitoring needs of the trial and agree on a monitoring plan.

15 HUMAN SUBJECTS

15.1 Institutional Review Board (IRB) Review and Informed Consent

This protocol and the informed consent document and any subsequent modifications will be reviewed and approved by the Institutional Review Board or Ethics Committee responsible for oversight of the study. Written informed consent will be obtained from the subject. The subject's assent must also be obtained if he or she is able to understand the nature, significance, and risks associated with the study. The informed consent will describe the purpose of the study, the procedures

to be followed, and the risks and benefits of participation. A copy of the consent form will be given to the subject.

15.2 Subject Confidentiality

All laboratory specimens, evaluation forms, reports, and other records will be identified by a coded number only to maintain subject confidentiality. All records will be kept in a locked file cabinet. All computer entry and networking programs will be done with coded numbers only. Clinical information will not be released without written permission of the subject, except as necessary for monitoring by the FDA, or the pharmaceutical sponsors.

15.3 Study Discontinuation

The study may be discontinued at any time by the investigators, the NIAID, the pharmaceutical sponsors, or the FDA.

16 PUBLICATION OF RESEARCH FINDINGS

Publication of the results of this trial will be governed by institutional policies. Any presentation, abstract, or manuscript will be made available for review by the Weill Medical College Office of Technology Development, the NIAID, and the pharmaceutical sponsors prior to submission.

17 BIOHAZARD CONTAINMENT

As the transmission of HIV and other blood-borne pathogens can occur through contact with contaminated needles, blood, and blood products, appropriate blood and secretion precautions will be employed by all personnel in the drawing of blood and shipping and handling of all specimens for this study, as currently recommended by the Centers for Disease Control and Prevention.

All infectious specimens will be sent using the ISS-1 SAF-T-PAK mandated by the International Air Transport Association Dangerous Goods Regulations-Packing Instruction 602. Please refer to individual carrier guidelines, e.g., FedEx, Airborne, for specific instructions.

As the transmission of HIV and other blood-borne pathogens can occur through contact with contaminated needles, blood, and blood products, appropriate blood and secretion precautions will be employed by all personnel in the drawing of blood and shipping and handling of all specimens for this study, as currently recommended by the Centers for Disease Control and Prevention.

All infectious specimens will be sent using the ISS-1 SAF-T-PAK mandated by the International Air Transport Association Dangerous Goods Regulations-Packing

Instruction 602. Please refer to individual carrier guidelines, e.g., FedEx, Airborne, for specific instructions.

18 REFERENCES

1. Gulick, R.M., J.W. Mellors, D. Havlir, J.J. Eron, C. Gonzalez, D. McMahon, D.D. Richman, F.T. Valentine, L. Jonas, A. Meibohm, E.A. Emini, and J.A. Chodakewitz. 1997. Treatment with indinavir, zidovudine, and lamivudine in adults with human immunodeficiency virus infection and prior antiretroviral therapy [see comments]. *N Engl J Med* 337, no. 11:734.
2. Hammer, S.M., K.E. Squires, M.D. Hughes, J.M. Grimes, L.M. Demeter, J.S. Currier, J.L. Eron, J.E. Feinberg, H.H. Balfour, L.R. Deyton, J.A. Chodakewitz, and M.A. Fischel. 1997. A controlled trial of two nucleotide analogues plus indinavir in persons with human immunodeficiency virus infection and CD4 cell counts of 200 per cubic millimeter or less. *N. Engl. J. Med.* 337:725.
3. Wong, J.K., M. Hezareh, H.F. Gunthard, D.V. Havlir, C.C. Ignacio, C.A. Spina, and D.D. Richman. 1997. Recovery of replication-competent HIV despite prolonged suppression of plasma viremia [see comments]. *Science* 278, no. 5341:1291.
4. Finzi, D., M. Hermankova, T. Pierson, L.M. Carruth, C. Buck, R.E. Chaisson, T.C. Quinn, K. Chadwick, J. Margolick, R. Brookmeyer, J. Gallant, M. Markowitz, D.D. Ho, D.D. Richman, and R.F. Siliciano. 1997. Identification of a reservoir for HIV-1 in patients on highly active antiretroviral therapy [see comments]. *Science* 278, no. 5341:1295.
5. Finzi, D., J. Blankson, J.D. Siliciano, J.B. Margolick, K. Chadwick, T. Pierson, K. Smith, J. Lisziewicz, F. Lori, C. Flexner, T.C. Quinn, R.E. Chaisson, E. Rosenberg, B. Walker, S. Gange, J. Gallant, and R.F. Siliciano. 1999. Latent infection of CD4+ T cells provides a mechanism for lifelong persistence of HIV-1, even in patients on effective combination therapy. *Nat Med* 5, no. 5:512.
6. Zhang, L., B. Ramratnam, K. Tenner-Racz, Y. He, M. Vesanen, S. Lewin, A. Talal, P. Racz, A.S. Perelson, B.T. Korber, M. Markowitz, D.D. Ho, Y. Guo, M. Duran, A. Hurley, J. Tsay, Y.C. Huang, and C.C. Wang. 1999. Quantifying Residual HIV-1 Replication in Patients Receiving Combination Antiretroviral Therapy. N. Engl J Med 1999, **340**:1605-1613.

7. Jubault, V., M. Burgard, E. Le Corfec, D. Costagliola, C. Rouziox, and J.-P. Viard. 1998. High rebound of plasma and cellular HIV load after discontinuation of triple combination therapy. *AIDS* 1998, **12**:2358-2359.

8. Staszewski, S., V. Miller, C. Sabin, A. Berger, A.M. Hill, and A.N. Phillips. 1998. Rebound of HIV-1 viral load after suppression to very low levels. *AIDS* 12, no. 17:2360.
9. Davey, R.T.J., N. Bhat, C. Yoder, T.-W. Chun, J.A. Metcalf, R. Dewar, V. Natarajan, R.A. Lempicki, J.W. Adelsberger, K.D. Miller, J.A. Kovacs,

M.A. Polis, R.E. Walker, J. Falloon, H. Masur, D. Gee, M. Baseler, D.S. Dimitrov, A.S. Fauci, and H.C. Lane. 1999. HIV-1 and T cell dynamics after interruption of highly active antiretroviral therapy (HAART) in patients with a history of sustained viral suppression. *Proc. Natl. Acad. Sci. U.S.A.*1999, **96**:15109-15114.

10. Callan, M.F., N. Steven, P. Krausa, J.D. Wilson, P.A. Moss, G.M. Gillespie, J.I. Bell, A.B. Rickinson, and A.J. McMichael. 1996. Large clonal expansions of CD8+ T cells in acute infectious mononucleosis. *Nat Med* 1996, **2**:906-911.
11. Wilson, J.D., G.S. Ogg, R.L. Allen, P.J. Goulder, A. Kelleher, A.K. Sewell, C.A. O'Callaghan, S.L. Rowland-Jones, M.F. Callan, and A.J. McMichael. 1998. Oligoclonal expansions of CD8(+) T cells in chronic HIV infection are antigen specific. *J Exp Med* 1998, **188**:785-790.

12. Murali-Krishna, K., J.D. Altman, M. Suresh, D.J. Sourdive, A.J. Zajac, J.D. Miller, J. Slansky, and R. Ahmed. 1998. Counting antigen-specific CD8 T cells: a reevaluation of bystander activation during viral infection. *Immunity* 1998, **8**:177-187.

13. Gallimore, A., A. Glithero, A. Godkin, A.C. Tissot, A. Pluckthun, T. Elliott, H. Hengartner, and R. Zinkernagel. 1998. Induction and exhaustion of lymphocytic choriomeningitis virus-specific cytotoxic T lymphocytes visualized using soluble tetrameric major histocompatibility complex class I-peptide complexes. *J Exp Med* 1998, **187**:1383-1393.

14. Mongkolsapaya, J., A. Jaye, M.F. Callan, A.F. Magnusen, A.J. McMichael, and H.C. Whittle. 1999. Antigen-specific expansion of cytotoxic T lymphocytes in acute measles virus infection. *J Virol* 1999, **73**:67-71.

15. Rosenberg, E.S., J.M. Billingsley, A.M. Caliendo, S.L. Boswell, P.E. Sax, S.A. Kalams, and B.D. Walker. 1997. Vigorous HIV-1-specific CD4+ T cell responses associated with control of viremia [see comments]. **]**. *Science* 1997, **278**:1447-1450.

16. Kasaian, M.T., K.A. Leite-Morris, and C.A. Biron. 1991. The role of CD4+ cells in sustaining lymphocyte proliferation during lymphocytic choriomeningitis virus infection. *J Immunol* 1991, **146**:1955-1963.

17. Matloubian, M., R.J. Concepcion, and R. Ahmed. 1994. CD4+ T cells are required to sustain CD8+ cytotoxic T-cell responses during chronic viral infection. *J Virol* 1994, **68**:8056-8063.

18. Cousens, L.P., J.S. Orange, and C.A. Biron. 1995. Endogenous IL-2 contributes to T cell expansion and IFN-gamma production during

lymphocytic choriomeningitis virus infection. *J Immunol* 1995, **155**:5690-5699.

19. Doherty, P.C. 1998. The numbers game for virus-specific CD8+ T cells. *Science* 1998, **280**:227.

20. Butz, E.A., and M.J. Bevan. 1998. Massive expansion of antigen-specific CD8+ T cells during an acute virus infection. *Immunity* 1998, **8**:167-175.

21. Smith, K., E. Jacobson, T. Sohn, D. Warren, R. Emert, and M. Giordano. 2000. In vivo assessment of antiviral reactivity in chronic HIV infection. *HIV Clinical Trials* 2000, **1**:16-22.

22. Jacobson, E.L., F. Pilaro, and K.A. Smith. 1996. Rational interleukin 2 therapy for HIV positive individuals: daily low doses enhance immune function without toxicity. *Proc Natl Acad Sci U S A* 1996, **93**:10405-10410.

23. . Pakker NG, Notermans DW, de Boer RJ, Roos MT, de Wolf F, Hill A, Leonard JM, Danner SA, Miedema F, Schellekens PT: **Biphasic kinetics of peripheral blood T cells after triple combination therapy in HIV-1 infection: a composite of redistribution and proliferation [see comments]**. *Nat Med* 1998, **4**:208-214.

24. 24. Smith KA: **T-cell growth factor**. *Immunol Rev* 1980, **51**:337-357.
.
25. Cantrell DA, Smith KA: **Transient expression of interleukin 2 receptors. Consequences for T cell growth**. *J Exp Med* 1983, **158**:1895-1911.

26. Cantrell DA, Smith KA: **The interleukin-2 T-cell system: a new cell growth model**. *Science* 1984, **224**:1312-1316.

27. Smith KA: **Interleukin-2: inception, impact, and implications**. *Science* 1988, **240**:1169-1176.

28. Ferrari G, Humphrey W, McElrath M, Excler J, Duliege A, Clements M, Corey L, Bolognesi D, Weinhold K: **Clade B-based HIV-1 vaccines elicit cross-clade cytotoxic T lymphocyte reactivities in uninfected volunteers.** *Proc Natl Acad Sci U S A* 1997, **94**: p1396-1401.

29. Clements-Mann M, Weinhold K, Matthews T, Graham B, Gorse G, Keefer M, McElrath M, Hsieh R, Mestecky J, Zolla-Pazner S, et al.: **Immune responses to human immunodeficiency virus (HIV) type 1 induced by canarypox expressing HIV-1MN gp120, HIV-1SF2 recombinant gp120, or both vaccines in seronegative adults. NIAID AIDS Vaccine Evaluation Group.** *J Infect Dis* 1998, **177**: p1230-1246.

30. Evans T, Keefer M, Weinhold K, Wolff M, Montefiori D, Gorse G, Graham B, McElrath M, Clements-Mann M, Mulligan M, et al.: **A canarypox vaccine expressing multiple human immunodeficiency virus type 1 genes given alone or with rgp120 elicits broad and durable CD8+ cytotoxic T lymphocyte responses in seronegative volunteers.** *J Infect Dis* 1999, **180**: p290-298.

31. Dolin R: **Human studies in the development of human immunodeficiency virus vaccines.** *J Infect Dis* 1995, **172**: p1175-1183.

32. Paoletti E, Tartaglia J, Taylor J: **Safe and effective poxvirus vectors--NYVAC and ALVAC.** *Dev Biol Stand* 1994, **82**:65-69.

33. Taylor J, Weinberg R, Longuet B: **Recombinant fowlpox virus including protective immunity in non-avian species**. *Vaccine* 1988, **6**:487-503.

34. Tartaglia J, Perkus M, Taylor J, Norton E, Audonnet J-C, Cox W: **NYVAC: a highly attenuated strain of vaccinia virus**. *Virology* 1992, **188**:217-232.

35. Taylor J, Meignier B, Tartaglia J, Languet B, Vander HJ, Franchini G, Trimarchi C, Paoletti E: **Biological and immunogenic properties of a canarypox-rabies recombinant, ALVAC-RG (vCP65) in non-avian species.** *Vaccine* 1995, **13**: p539-549.

36. Ferrari G, Berend C, Ottinger J, Dodge R, Bartlett J, Toso J, Moody D, Tartaglia J, Cox W, Paoletti E, et al.: **Replication-defective canarypox (ALVAC) vectors effectively activate anti-human immunodeficiency virus-1 cytotoxic T lymphocytes present in infected patients: implications for antigen-specific immunotherapy.** *Blood* 1997, **90**: p2406-2416.

37. Jin X, Ramanathan M, Barsoum S, al. e: **Safety and Immunigenicity of ALVAC vCP1452 and Recombinant gp160 in Newly Human Immunodeficiency Virus Type-1-Infected Patients Treated with Prolonged Highly Active Antiretroviral Therapy.** *J. Virol.`* 2002, **76**:2206-2216.

38. Smith KA, Jacobson EL, Emert R, Giordano M, Kovacs E, Mumneh N, Pilaro F, Sohn T, Warren D: **Restoration of immunity with interleukin 2 therapy**. *The Aids Reader* 1999, **9**:563-572.

39. . Lalezari J, Beal J, Ruane P, Cohen C, Jacobson E, Sundin D, Leong W, Raffanti S, Wheeler D, Anderson R, et al.: **Low dose daily subcutaneous interleukin 2 in combination with highly active antiretroviral therapy in HIV+ patients: a randomized, controlled trial.** *HIV Clinica Trials* 2000, **1**:1-15.

40. Kovacs JA, Baseler M, Dewar RJ, Vogel S, Davey RT, Jr., Falloon J, Polis MA, Walker RE, Stevens R, Salzman NP, et al.: **Increases in CD4 T lymphocytes with intermittent courses of interleukin- 2 in patients with**

human immunodeficiency virus infection. A preliminary study [see comments]. *N Engl J Med* 1995, **332**:567-575.
