# Supplementary material for: Immunotherapy with Canarypox Vaccine and Interleukin-2 for HIV-1 Infection: Termination of a Randomized Trial
Source: PLoS Clin Trials. 2007 Jan 26;2(1):e5. doi: 10.1371/journal.pctr.0020005 (PMC1783674; doi:10.1371/journal.pctr.0020005)
Supplement: Text S1 — (27 KB DOC) [file pctr.0020005.sd003.doc]

### Supporting Information

Laboratory Evaluations:

HIV RNA Concentrations

Plasma HIV RNA concentrations ([HIV]) were monitored by the NYPHL using the COBAS Amplicore HIV-1 Monitor Test, v 1.5™ (Roche Diagnostics, Nutley, NJ), which demonstrates reliable quantification of low plasma [HIV] with a detection rate of greater than 95% at ≥ 50 HIV RNA copies/mL.

Lymphocyte Subset Concentrations

Lymphocyte subsets were monitored by the NYPHL using a FACSCalibur™ flow cytometer according to the manufacturer’s instructions (BD Biosciences, San Jose, CA; BD). Three-color flow cytometry was performed using anti-CD45-PerCP to identify lymphocytes, anti-CD3-FITC to identify T cells, and anti-CD4-PE (Th), anti-CD8-PE (Tc), anti-CD19-PE (B cell), and anti-CD16+CD56-PE (NK cell) to identify the various lymphocyte subsets. All antibodies were from BD. A simultaneous complete blood cell and differential cell count was used to obtain the circulating absolute lymphocyte concentration, to calculate the absolute concentrations of each lymphocyte subset. The CD4^+^ and CD8^+^ T cell concentrations were then used to calculate the concentrations of HIV-reactive T cells, expressed as cells/mL (instead of the routine cells/μL), so as to be consistent with the plasma [HIV], which is traditionally expressed in HIV mRNA copies/mL. To ensure an accurate baseline value of the concentration of circulating lymphocyte subsets for comparison with values obtained during the study, the mean of 3 separate weekly baseline determinations was calculated and was expressed as 100%.

HIV-Specific Cytokine Producing Lymphocyte Precursor Concentrations

HIV-reactive T cells were quantified by the Immunology Division Research Laboratory using a FACSCalibur™ flow cytometer via a FastImmune™ method developed by BD for measuring Ag-specific cytokine production in fresh blood samples[26,27,28]. Heparinized blood, first incubated overnight at 20° C, was then activated for 6 hours with a pool of 15-mer peptides overlapping every 11 amino acids predicted by the entire p55 *gag* HIV gene sequence, in the presence of costimulatory antibodies reactive with CD28 and CD49b, as well as Brefeldin-A (10 μg/mL) to block protein secretion. Each peptide in the pool was added at a final concentration of 1 μg/mL. Following a 6-hour incubation at 37° C, the erythrocytes were lysed using 9 mL FACSLysing™ solution/mL blood and the cells were frozen at -80° C. After thawing, the cells were washed with Phosphate Buffered Saline (PBS) and permeabilized using FACS™ Permeabilizing Solution (0.5 mL), after which the cells were stained with fluorochrome-labeled monoclonal antibodies (MoAb) reactive with CD3, CD4, CD8, and CD69 to identify activated T cell subsets, and IL2, Interferon-gamma (IFNγ), and Tumor Necrosis Factor-alpha (TNFα) to quantify T cells capable of producing these cytokines. BD specially formulated fluorochrome- labeled MoAb cocktails: cytokine FITC (IFNγ, TNFα or IL2)/CD69-PE/CD4-PerCP or CD8-PerCP/CD3-APC.

Samples were analyzed by four-color flow cytometry. At least 50,000 events were acquired within the lymphocyte gate, determined by typical forward and side light scattering. At least 10,000 events (30,000-50,000 events for the majority of samples) were acquired within the T cell gate, which was determined by the expression of CD3 and CD4 or CD8 molecules. HIV p55*gag*-specific immune responses were quantified as the % of CD69^+^cytokine+ (IFNγ, TNFα and IL2) CD8^+^ or CD4^+^ T cells at various time points. CellQuest™ Pro software was used for the acquisition and analysis. Unstimulated samples and fluorochrome-labeled isotype controls were used to set the quadrants for analysis of samples, and the % of cells in the unstimulated samples were subtracted from the stimulated samples to determine the % of HIV p55 *gag-*specific cells. The concentration of cells reactive with the HIV p55*gag* pool was then calculated from the concentrations of CD4^+^ and CD8^+^ T cells (CD4/8 cells/mL) measured at the NYPHL according to the formula: (%CD69^+^cytokine^+^) / (100 × CD4/8 cells/mL).
